# Microsecond motions probed by near-rotary-resonance *R*_1ρ_^15^N MAS NMR experiments: the model case of protein overall-rocking in crystals

**DOI:** 10.1007/s10858-018-0191-4

**Published:** 2018-05-30

**Authors:** Alexey Krushelnitsky, Diego Gauto, Diana C. Rodriguez Camargo, Paul Schanda, Kay Saalwächter

**Affiliations:** 1 Martin-Luther-Universität Halle-Wittenberg, Halle, Germany; 2 Institut de Biologie Structurale (IBS), Grenoble Cedex 9, France; 3 Technische Universität München, Garching, Germany; 4 Present Address: Wren Therapeutics Ltd., Cambridge, UK

**Keywords:** NMR relaxation, Protein crystals, Molecular dynamics, Rotary resonance, Magic angle spinning, GB1, SH3 domain, Ubiquitin

## Abstract

Solid-state near-rotary-resonance measurements of the spin–lattice relaxation rate in the rotating frame (*R*_1ρ_) is a powerful NMR technique for studying molecular dynamics in the microsecond time scale. The small difference between the spin-lock (SL) and magic-angle-spinning (MAS) frequencies allows sampling very slow motions, at the same time it brings up some methodological challenges. In this work, several issues affecting correct measurements and analysis of ^15^N *R*_1ρ_ data are considered in detail. Among them are signal amplitude as a function of the difference between SL and MAS frequencies, “dead time” in the initial part of the relaxation decay caused by transient spin-dynamic oscillations, measurements under HORROR condition and proper treatment of the multi-exponential relaxation decays. The multiple ^15^N *R*_1ρ_ measurements at different SL fields and temperatures have been conducted in 1D mode (i.e. without site-specific resolution) for a set of four different microcrystalline protein samples (GB1, SH3, MPD-ubiquitin and cubic-PEG-ubiquitin) to study the overall protein rocking in a crystal. While the amplitude of this motion varies very significantly, its correlation time for all four sample is practically the same, 30–50 μs. The amplitude of the rocking motion correlates with the packing density of a protein crystal. It has been suggested that the rocking motion is not diffusive but likely a jump-like dynamic process.

**Electronic supplementary material:**

The online version of this article (10.1007/s10858-018-0191-4) contains supplementary material, which is available to authorized users.

## Introduction

Spin–lattice relaxation in the rotating frame is a powerful NMR method used to obtain quantitative information on molecular dynamics in the microsecond timescale. During the recent decade, measurements of the ^15^N rotating-frame relaxation rate (*R*_1ρ_ = 1/*T*_1ρ_) have been widely applied in the solid-state NMR studies of protein dynamics. Combination of fast magic angle spinning (MAS) and/or partial deuteration of proteins enable obtaining well resolved ^1^H–^15^N correlation spectra and, as a consequence, capability to measure site-specific relaxation rates with a negligible spin–spin contribution to the incoherent relaxation (Krushelnitsky et al. 2010; Zinkevich et al. 2013; Good et al. 2014, 2017; Lamley et al. 2015a, b; Ma et al. 2015; Smith et al. 2016; Kurauskas et al. 2016, 2017; Saurel et al. 2017; Lakomek et al. 2017; Gauto et al. 2017). The ability to vary the spin-lock field strength from < 1 to 40–50 kHz enables covering a wide frequency range of dynamics, and the simultaneous analysis of both the chemical-exchange contribution to *R*_1ρ_ and the dipole/CSA relaxation mechanisms (Ma et al. 2014; Lamley et al. 2015b) provides abundant data that characterize protein dynamics in much detail. The concept of excited states of proteins (Mulder et al. 2001; Korzhnev and Kay 2008), that is highly relevant to mechanisms of protein biological function, stresses the importance of *R*_1ρ_ experiments since they can effectively monitor the exchange between the ground and excited states on the microsecond timescale.

The *R*_1ρ_ rate constant in static samples is proportional to the spectral density at the spin-lock frequency. When measured under MAS, *R*_1ρ_ due to the heteronuclear dipolar and CSA relaxation mechanisms is proportional to the following combination of the spectral-density functions:

1 $$R_{{1\rho }}^{{}} \propto \left( {J({\omega _{SL}} - 2{\omega _{MAS}})+2J({\omega _{SL}} - {\omega _{MAS}})+2J({\omega _{SL}}+{\omega _{MAS}})+J({\omega _{SL}}+2{\omega _{MAS}})} \right)$$

where *ω*_*MAS*_/2π and *ω*_*SL*_/2π are the MAS and spin-lock frequencies, respectively (Haeberlen and Waugh 1969; Kurbanov et al. 2011). The dependence on the spectral density at the frequency difference between spin-lock and MAS frequencies makes it theoretically possible to measure very slow motions. If *ω*_*MAS*_ ~ *ω*_*SL*_ or 2*ω*_*MAS*_ ~ *ω*_*SL*_ then, according to Eq. (1), one can measure even the zero-frequency limit of the spectral density function *J*(0). In practice, however, it is not feasible to benefit from this advantage since the conditions *ω*_*MAS*_ = *ω*_*SL*_ and 2*ω*_*MAS*_ = *ω*_*SL*_ correspond to the rotary resonance (RR) phenomenon (Oas et al. 1988; Levitt et al. 1988), at which the dipolar and CSA interactions are recoupled, and the spins thus evolve under these interactions. Spin evolution at these conditions, therefore, is governed by these coherence mechanisms, rather than molecular motions. Rotary resonance is not considered in the Redfield theory and thus, it cannot be taken into account in the data analysis in a quantitative manner. Although the exact *J*(0) measurement is not possible, still sampling the slow dynamics with the help of *R*_1ρ_ experiments at small difference between *ω*_*MAS*_ and *ω*_*SL*_ is feasible (Zinkevich et al. 2013; Ma et al. 2014; Kurauskas et al. 2017).

The aim of this work is to delineate the practical challenges and limitations of MAS *R*_1ρ_ measurements and data analyses in the vicinity of the RR conditions and to demonstrate the capability of these experiments to study slow molecular dynamics. We use numerical computer simulations and ^15^N MAS *R*_1ρ_ measurements of four different protein samples. Among the problems considered in this study are interfering spin-dynamics effects, the homonuclear rotary resonance (HORROR) condition, and the shape of the relaxation decay. We finally demonstrate the potential of *R*_1ρ_ experiments by studying the rocking motion of proteins in solid environment.

## Materials and methods

### Numerical simulations

Spin dynamics simulations were conducted using a home-written code described earlier (Saalwächter and Fischbach 2002). This program is based on a finite-step integration of the Liouville-von-Neumann equation, representing a finite number of (rotational) states accessed by a dynamic process by a vector of density matrices and associated Hamiltonians. The molecular dynamics is implemented in separate mixing steps among the density matrices on the basis of a pre-defined exchange matrix, which alternate with the quantum-mechanical evolution using single-site propagators. This approach was demonstrated to provide quantitative results once sufficiently small time steps are used, and avoids the exceedingly large dimension of the full Liouvillian. In the current work, the simulations always include the simplest motional model: jumps between two equivalent sites with different orientations of the N–H vector.

### Samples

In this work four different microcrystalline protein samples were studied. These were the α-spectrin SH3 domain, GB1, and ubiquitin in two crystal polymorphs. The SH3 domain was purified and crystallized in Bernd Reif’s laboratory (FMP Berlin) according to a protocol described in (Chevelkov et al. 2006). The crystalline GB1 sample was purchased from Giotto Biotech. The sample was prepared according to the protocol described in (Franks et al. 2005). The only modification of the protocol was a usage of a mixture of 80% deuterated and 20% protonated solvents (both organic solvents and water buffer) instead of 100% protonated ones, since in the latter case the lines were too wide at 20 kHz MAS. Two different polymorphs of ubiquitin were used, corresponding to the previously described “MPD-ub” and “cubic-PEG-ub” forms (Ma et al. 2015). All proteins were uniformly ^15^N,^2^H-labeled by recombinant protein production in D_2_O-based minimal medium, and the exchangeable hydrogens were exchanged to ^1^H by placing the protein in a mixture of H_2_O and D_2_O-based buffer before crystallization. The extent of the proton back-exchange was 20% for SH3 domain and GB1 and 30% for ubiquitin. This high deuteration level enables high-resolution proton-detected ^1^H–^15^N experiments (Chevelkov et al. 2006) and negligible spin–spin contribution to *R*_1ρ_ (Krushelnitsky et al. 2010, 2014).

### NMR experiments

**Fig. 1:**
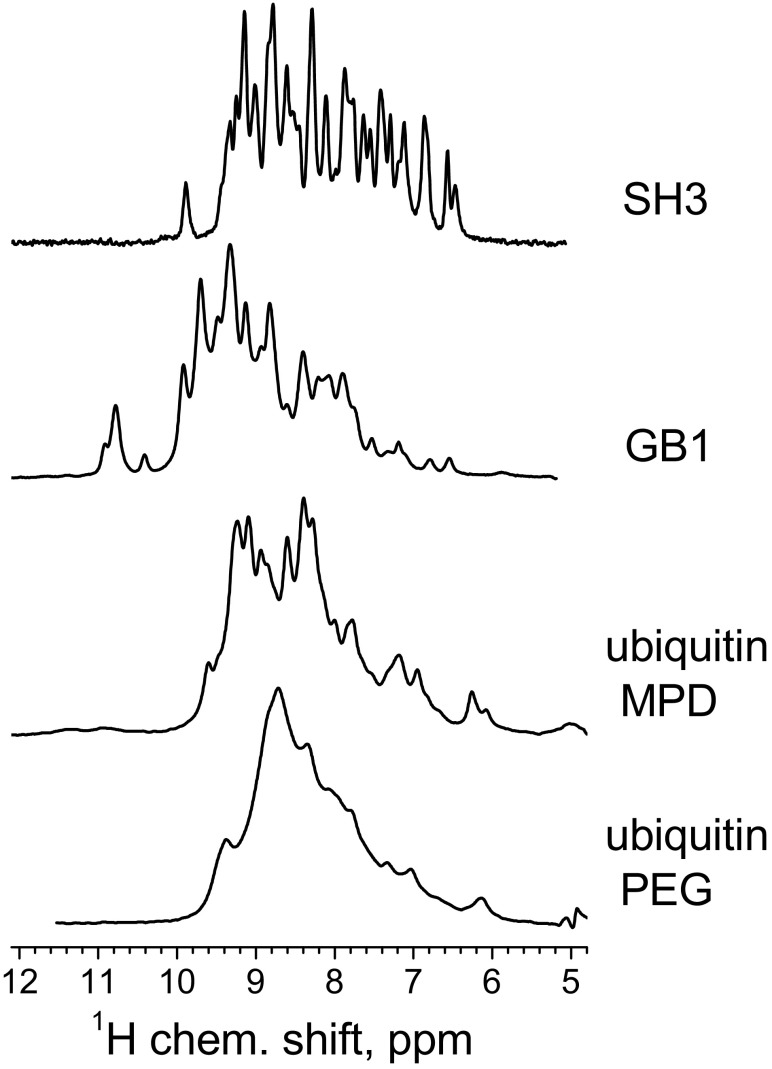
Proton-detected 1D spectra at room temperature of the four samples used in this study. For plotting the relaxation decays, in all cases the integral under the entire spectrum was used

The SH3 domain and the GB1 protein were measured at Halle University, while the two ubiquitin samples were measured at the IBS Grenoble. In both cases measurements were performed on a Bruker 600 MHz spectrometer. 3.2 and 1.3 mm MAS probes were used with MAS rates 20 and 40 kHz in Halle and Grenoble, respectively. *R*_1ρ_ decays were measured using routine double-CP (i.e., CP from protons to nitrogens and then back to protons) pulse sequences with proton detection of the signal described in (Krushelnitsky et al. 2010; Ma et al. 2014). In the middle of the ^15^N spin-lock pulse, a proton π-pulse was applied in order to exclude the dipole-CSA cross-correlation effect (Kurauskas et al. 2016). In this work one-dimensional proton spectra at different durations of the ^15^N spin-lock pulse were measured and then the relaxation decay was obtained from the integrated signal of the entire amide band of the ^1^H spectrum. One-dimensional spectra of the four proteins are shown in Fig. 1. While this 1D approach excludes the possibility to characterize protein dynamics site-specifically, it enables conducting a large number of experiments (relaxation measurements with comparably many relaxation delays at different spin-lock field strengths and temperatures, see below) within reasonable time limits. The relaxation experiments were conducted at temperatures 13, 21.5 and 29 °C for the SH3 domain and GB1, and 3, 15 and 27 °C for the two ubiquitin samples. The temperature calibration was performed using the MAS rotor with ~ 5 μl of methanol, the calibration accuracy was ± 1.5 °C.

## Results and discussion

### Experimental methodology

#### Initial oscillations

**Fig. 2:**
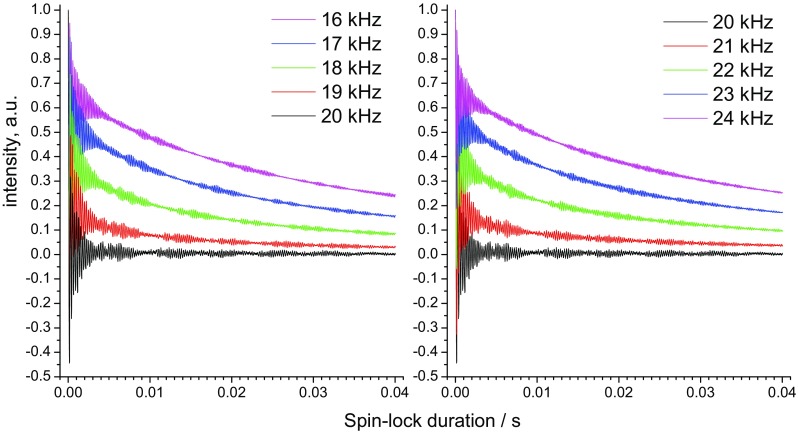
Numerically simulated ^15^N *R*_1ρ_-relaxation decays for ^15^N–^1^H spin pair undergoing two-site jumps with an angular amplitude 20° and an exchange rate of 500 s^−1^ (^15^N–^1^H dipolar coupling 11.5 kHz, CSA interaction not included). The simulations were performed at 20 kHz MAS and different on-resonance spin-lock fields at different RF amplitude as indicated by different colours. In all cases the first point of the decays at t = 0 has an amplitude of unity

**Fig. 3:**
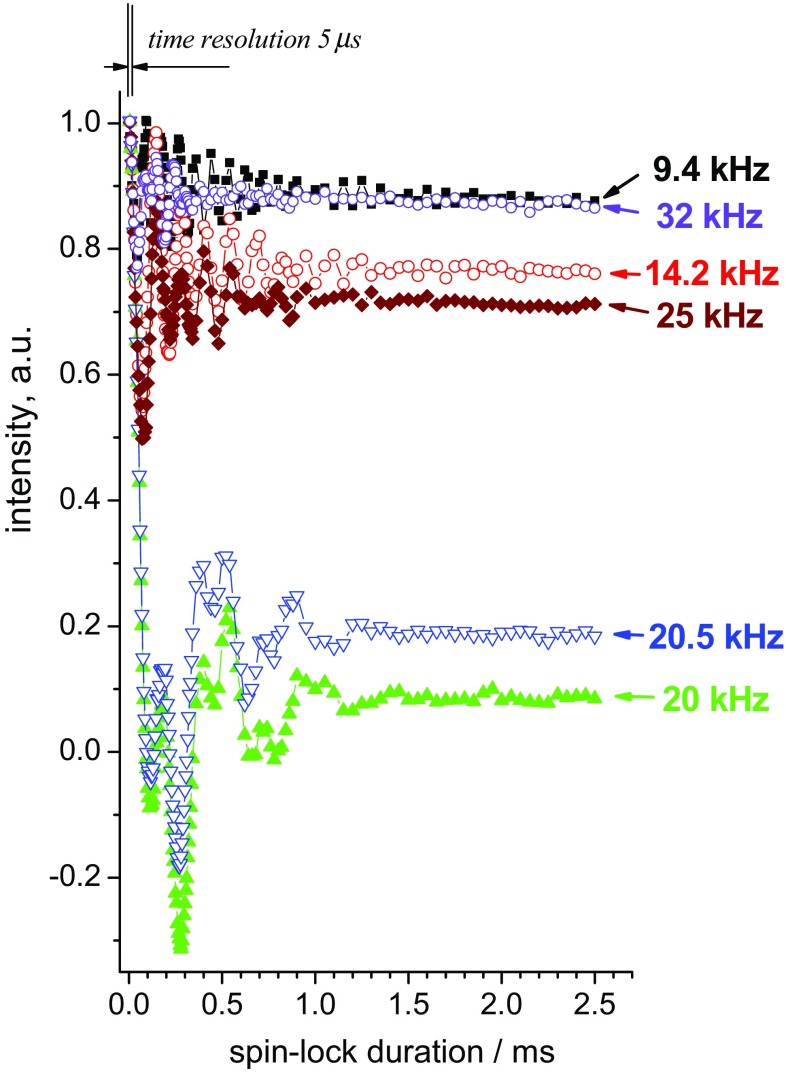
Experimentally measured ^15^N *R*_1ρ_-relaxation decays (initial part) of the integral signal in GB1 protein at 20 kHz and different spin-lock fields as indicated. All decays were normalized to unity at the initial point at 5 μs

Figures 2 and 3 present numerically simulated and experimentally measured *R*_1ρ_ relaxation decays at different spin-lock fields, respectively. The initial part of the decays always contains coherent transient oscillations. The physical nature of such oscillations in *R*_1ρ_ experiments has been described a long time ago (VanderHart and Garroway 1979). The exact shape of these oscillations depends on the MAS rate, strength of the dipolar and CSA interactions and spin lock frequency. To the best of our knowledge, detailed theoretical treatments of this process are not available, and we discuss the transient oscillations only in a phenomenological manner. We note, however, that these oscillations contain no information about molecular dynamics and they do not affect the shape of the relaxation decay at longer delays, which are the focus of this work. We also note that the physical origin of these oscillations and spin–spin (coherent) contribution to the relaxation rate is not the same (VanderHart and Garroway 1979). In many previous experimental studies, the presence of these oscillations has been overlooked, because they can be observed only by measuring multiple relaxation delays with a small time step over the initial part of the decay, which is hardly possible with a typical number (5–15) of relaxation delays in 2D *R*_1ρ_ experiments. The oscillations themselves are not informative for dynamic investigations, but including a relaxation delay that falls within these oscillations can lead to artefacts when attempting to extract relaxation rate constants. In fact, these oscillations play the role of a “dead time” in the *R*_1ρ_ experiments; thus before running the relaxation measurements it is advisable to determine its duration.

**Fig. 4:**
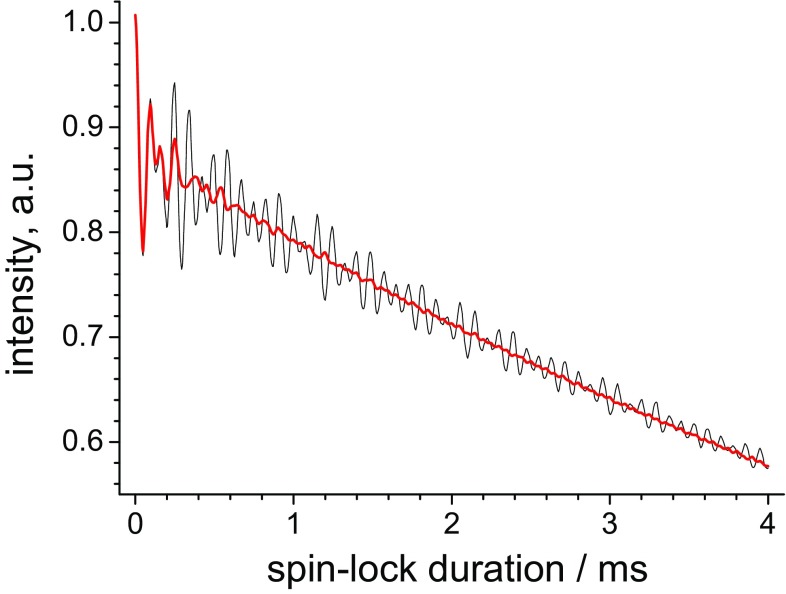
Simulated *R*_1ρ_ decays for the ^15^N–^1^H pair undergoing two-site jumps at a single spin-lock field of 31.5 kHz (black line) and superposition of the decays simulated at *B*_1_-fields from 30 to 33 kHz with a step of 250 Hz (red line). MAS rate 20 kHz

The time span of the initial oscillations is rather different in the simulations and experiment: in the case of experimental measurements the oscillations decay much faster, typically within ca. 2–3 ms, while in simulations they extend to ca. 10 ms (compare Figs. 2, 3). This observation has important experimental consequences and is in practice beneficial, because oscillations as long as those observed in simulations would hamper precise measurements of the relaxation rates. We ascribe the shorter life time of the oscillations in experiments to *B*_1_-field inhomogeneity. The frequency of these oscillations (at least the fundamental component; they are not purely harmonic) depends on the difference between spin-lock and MAS frequencies. Thus, *B*_1_-inhomogeneity causes a superposition of frequencies which in turn renders the “dead time” shorter. This is demonstrated in Fig. 4 by comparing the relaxation decays simulated at a single (ideally homogeneous) and 10%-inhomogeneous *B*_1_-field. This level of inhomogeneity is a realistic estimation for solid-state MAS probes (Haller and Schanda 2013; Tosner et al. 2017; Nagashima et al. 2017). This comparison demonstrates that a moderate level of *B*_1_ inhomogeneity is actually advantageous. Since the field inhomogeneity can be different for different probes (coils) and samples, the “dead time” in *R*_1ρ_-experiments can also be somewhat different, however, we believe it is always in the range of few ms. Of note, the *B*_1_-inhomogeneity renders the exact theoretical description of the initial transient oscillations rather difficult for practical purposes, since the *B*_1_-profile over a sample is not known in general case and is difficult to measure.

It is worth mentioning that the *B*_1_-field inhomogeneity can be a limiting factor for the minimal value of the difference between the spin-lock and MAS frequencies. If this difference is smaller than the width of the distribution of the spin-lock frequencies, then the quantitative analysis of the relaxation data using Eq. (1) might be ambiguous for the same reason—the exact shape of the *ω*_*SL*_ distribution in general case is not known. However, the more significant limiting factor for this difference is a sensitivity problem that is discussed below.

#### Initial decay amplitude at different spin-lock fields and the HORROR condition

Figures 2 and 3 demonstrate that the amplitude of the “useful” incoherent relaxation component of the decays decreases upon approaching the RR condition, while the “dead time” remains practically the same. Therefore, the RR condition seems to mainly affect the signal-to-noise ratio of the relevant later part of the decay but does not lead to serious systematic variation in the measured *R*_1ρ_. This should now be demonstrated in more detail.

**Fig. 5:**
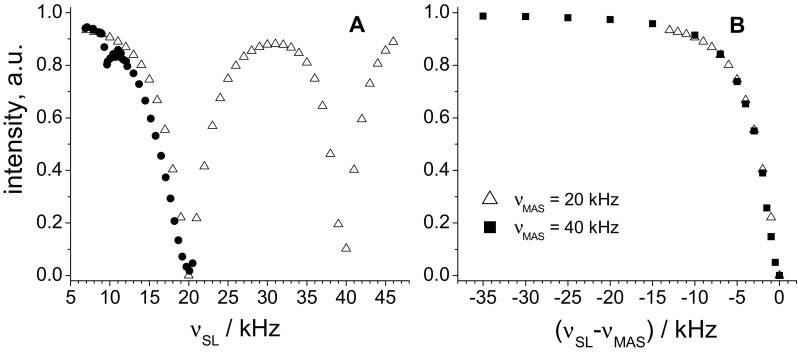
**a** Experimental (solid circles) and simulation (open triangles) amplitude of the relaxation decay at 20 kHz MAS as a function of the spin-lock field. The experimental dependence was arbitrarily normalized in order to get a coincidence with the simulation dependence at low spin-lock fields. **b** The amplitudes of the relaxation signal simulated at MAS rates 20 and 40 kHz plotted as a function of the difference between the spin-lock and MAS frequencies. Experiments were conducted with the GB1 sample. For the simulations, the model of 2º-jumps with the jump rate 5000 s^−1^ was used

Figure 5a presents the experimental and simulation dependencies of the amplitude of the decay as a function of the spin-lock field. Experimentally only one point at spin-lock duration 3 ms was measured, being the point where the initial oscillations have practically vanished. In simulations, the amplitude of the decay was determined by a single-exponential fit of the initial (4–6 ms) part of the decay. This fit provides the middle line of the oscillating decays since upper and lower half-periods of the oscillations compensate each other, and its extrapolation to zero time determines the amplitude plotted in Fig. 5. The coincidence between the simulated and experimental dependences is quite good. This figure demonstrates that *R*_1ρ_ can be measured almost at all spin-lock fields except the very narrow range around the rotary resonance points where the usable signal decreases drastically. Thus, if very slow dynamics is not the aim of the study, it is advisable to keep the difference between MAS and spin-lock frequencies as high as possible (at least more than 3–5 kHz) for a better signal-to-noise ratio. Otherwise, one should seek for a compromise between the small frequency difference and the signal amplitude, which can be specific for different samples and experimental conditions. Figure 5b shows that the relaxation signal amplitude depends only on the difference between the spin-lock and MAS frequencies, but the absolute values of these frequencies do not matter.

**Fig. 6:**
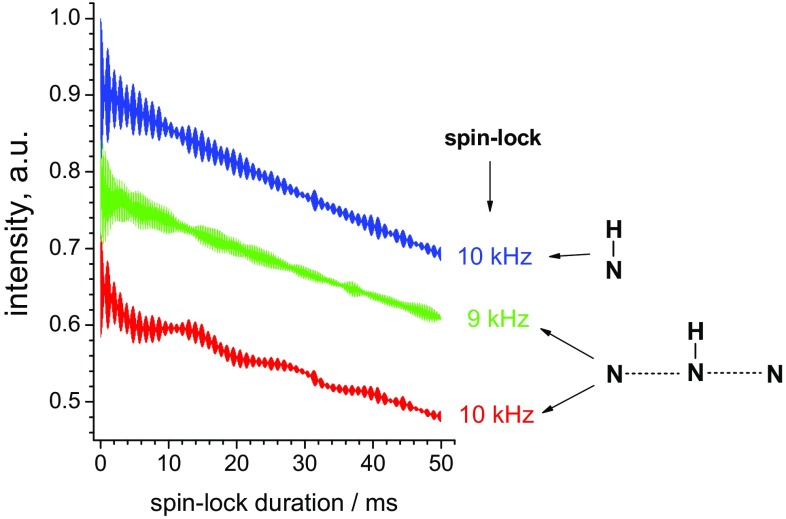
Three *R*_1ρ_ decays simulated for 20 kHz MAS, different spin-lock fields and different spin systems (indicated in the figure). ^15^N–^1^H distance in both cases was 1.02 Å, ^15^N–^15^N distances in 4-spins structure was 2.8 Å, which corresponds to the typical distance between backbone nitrogen atoms of neighbouring residues in a protein. In all cases two-site jumps of the ^15^N–^1^H bond with angular amplitude 5° were considered at a jump rate of 5000 s^−1^

A very interesting feature in the experimental dependence in Fig. 5 is the drop of the amplitude at spin-lock 10 kHz, which is exactly half of the MAS frequency. This behaviour cannot be reproduced in the simulations of the ^15^N–^1^H pair. However, when simulating the 4-spins structure shown in Fig. 6, the shape of the relaxation decay at 10 kHz becomes more complicated, revealing additional slower oscillations of the amplitude. This corresponds to the HORROR condition (Nielsen et al. 1994) which of course arises from homonuclear dipolar ^15^N–^15^N interactions. The ^15^N–^15^N interaction in the 4-spins structure (Fig. 6) is rather weak (56 Hz), however, this is sufficient for an appreciable distortion of the shape of the relaxation decay. Thus, in spite of the fact that the dominating relaxation mechanisms in ^15^N *R*_1ρ_ experiments in proteins are the heteronuclear ^1^H–^15^N dipole–dipole and ^15^N CSA mechanisms, the HORROR condition should be also avoided as well as rotary resonance condition, otherwise the observed decay contains also the coherent evolution due to the dipolar ^15^N–^15^N (at the HORROR condition) and the ^1^H–^15^N dipole–dipole and ^15^N CSA mechanisms.

#### Non-exponential shape of the relaxation decays

**Fig. 7:**
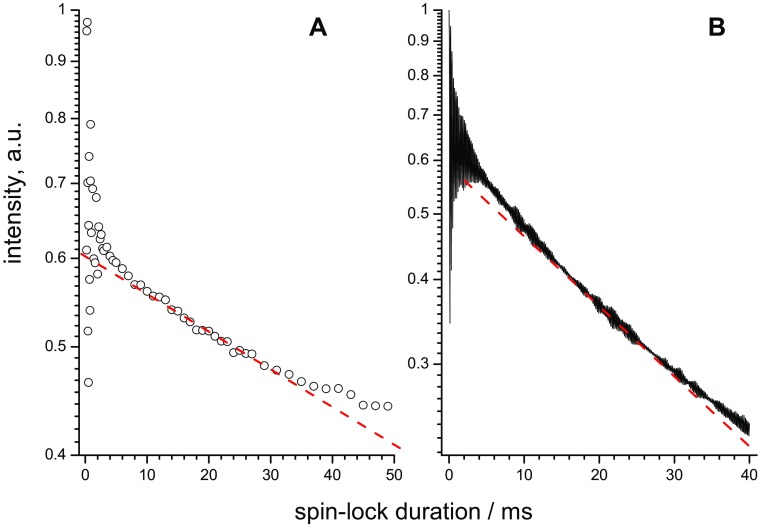
Typical examples of experimental (**a**) and simulated (**b**) *R*_1ρ_ decays on a semi-logarithmic scale. Dashed red lines indicate single-exponential decays for comparison. Experiment: SH3 sample, MAS 20 kHz, spin-lock field 15 kHz; simulations: jump amplitude 20°, rate 500 s^−1^

Figure 7 presents typical examples of the experimental and simulated *R*_1ρ_ relaxation decays on a semilogarithmic scale. It is seen that the decays are not straight lines, i.e. they cannot be described by single-exponential functions. Hence, there is a distribution of relaxation rate constants, which manifests the inhomogeneity of the spin system. We stress that this inhomogeneity is observed not only for the integral signal of a protein (which should be since a protein is a rather inhomogeneous structure with a relatively wide distribution of relaxation rates for different sites, see below) but also for a single ideal ^15^N–^1^H spin pair, the latter being relevant for the analysis of the site-specific relaxation decays. The reason of the inhomogeneity in the case of isolated ^15^N–^1^H pair is a powder averaging: the internuclear dipolar coupling and hence the relaxation rate depend on the orientation of the internuclear vector with respect to the *B*_0_ field. Thus, in solids the relaxation decays, both *R*_1_ and *R*_1ρ_, are always non-exponential if powder averaging is relevant and if there is no fast spin diffusion (Torchia and Szabo 1982; Giraud et al. 2005; Schanda and Ernst 2016). In static solids, the decays are strongly multi-exponential; MAS causes a partial averaging of the dipolar couplings, which is still not complete, and the decays remain non-exponential although this is not always clearly seen in the experiments due to the insufficient signal-to-noise ratio. The exact shape of the relaxation decay cannot be calculated for a general case since it depends on the angular amplitude of motion.

**Fig. 8:**
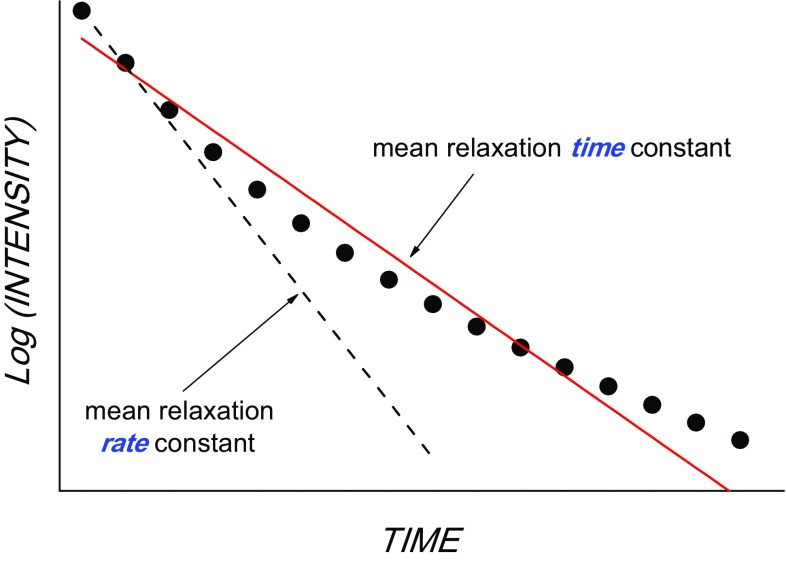
Schematic presentation of the multi-exponential experimental relaxation decay (circles), initial slope (dashed line) and fitting the whole decay using a single-exponential function (red solid line)

For a quantitative analysis in terms of the motional correlation function, one usually employs a model-free approach or its modifications in order to estimate a single relaxation parameter from such a non-exponential relaxation decay. This can be done in two ways: one may define the mean relaxation time constant or the mean relaxation rate constant. The former is provided by fitting the whole decay using the single-exponential fitting function, while the latter is represented by the initial slope of the decay (see Supp. Info to ref. Krushelnitsky et al. 2014), as demonstrated in Fig. 8. While both procedures are mathematically correct, only the mean relaxation rate constant has a physical meaning, as shown a long time ago (Kalk and Berendsen 1976). The relaxation rate constant is directly proportional to the spectral density function *R*_1,1ρ_ ~ *J*(ω), hence the mean relaxation rate constant is proportional to the mean spectral density function: <*R*_1,1ρ_> ~ <*J*(ω)>. This is not the case for the mean relaxation time constant. The correlation function obtained from the analysis of the initial slope of the relaxation decays is formally identical to that obtained from solution-state relaxation times analyses (Torchia and Szabo 1982).

Although these issues have been known for a long time, solid-state relaxation decays have often been analyzed in terms of mean relaxation time (not rate) constants, which in some cases may lead to incorrect conclusions, one of such examples is described in (Smith et al. 2018).

Practically, the initial slope can be determined from fitting the decay with a sum of few exponential functions or using any phenomenological distribution function for the relaxation rates. From such fittings, the mean relaxation rate can be readily defined. According to our experience, fitting functions with a minimal number of independent parameters such as a double exponential (two *R*_1ρ_, one relative amplitude, amplitude normalization coefficient) or a decay based upon a single-mode log-normal rate distribution (one median *R*_1ρ_, one distribution width parameter, amplitude normalization coefficient) are sufficient practically in all cases (Roos et al. 2015). Some practical hints for the proper analysis of non-exponential decays are described in ESM.

It must be mentioned that in the case of the *R*_1ρ_ experiments, the initial slope of the decays is not seen due to the initial oscillations considered above, i.e. “dead time”. At the moment we are not in a position to suggest a truly robust and reliable way to decompose oscillations and relaxation contributions at the beginning of the decay. Thus, the correct quantitative analysis of the non-exponential *R*_1ρ_ decays is only possible when the “dead time” is considerably shorter than the inverse value of the relaxation rate, enabling a stable minimal-parameter fit as discussed above at times beyond the dead time.

### Whole-protein rocking motion

The 1D-integral experiments discussed above do not allow obtaining detailed site-specific information on slow conformational protein dynamics. On the other hand, there is a kind of molecular motion for which the site-specific spectral resolution is not critical, which is the overall rocking motion of proteins in a solid environment, as reported recently both experimentally and computationally (Ma et al. 2015; Lamley et al. 2015b; Kurauskas et al. 2017). Such a global process assumes that all parts of a protein undergo the same motion and the correlation functions of all N–H bonds have a component with the same correlation time. The parameters of this common component can be obtained from 1D experiments and site-specific resolution for this is not necessary. The amplitude of this overall motion for different N–H bonds can be however different. For example, if the rocking motion is a restricted rotation around an axis then the amplitude (order parameter) depends on the angle between this axis and N–H bond. Thus, the rocking motion amplitude obtained from 1D experiments is a mean amplitude over all N–H bonds. As discussed below, the overall motion in general case can be overlapped with the internal conformational motion that has similar time scale. In this case, the unambiguous discrimination between these two types of molecular mobility from 1D data is impossible and site-specific measurements are necessary.

Previous analyses have shown that protein rocking occurs on the microsecond timescale (Lamley et al. 2015b; Kurauskas et al. 2017), thus near-rotary-resonance *R*_1ρ_ measurements should provide critically important information on the parameters of the rocking motion. In this part of the work we apply ^15^N *R*_1ρ_ experiments for a comparative study of the rocking motion in a set of four different solid protein samples, taking into account all the methodological problems discussed above. Since in all four cases the experiments and the data analyses were performed in the same manner, the results can be compared directly.

Interpreting relaxation measurements in terms of molecular motion is generally challenged by the fact that the spectral density function is sampled usually only at few frequencies. When only small number of relaxation parameters are measured, the fitted time constants and amplitudes may be subject to a large uncertainty (Smith et al. 2017). To resolve, at least partially, these ambiguities in determining motional time scales, we use relaxation measurements at multiple temperatures. Even when measured within a narrow temperature range, the temperature dependence of a relaxation rate, i.e. its slope—even the sign of the slope (positive or negative)—is very informative for the determination of the motional correlation time. Varying both the RF field strength and the temperature and analysing all the data simultaneously renders the set of the relaxation data effectively “two-dimensional”, which significantly improves the accuracy and precision of the fit results. The 1D approach allowed the collection of such an extensive data set at multiple RF field strengths, three different temperatures and four different protein samples.

Below we present the analysis of the *R*_1ρ_ rates measured at a wide range of differences (*ω*_*MAS*_ − *ω*_*SL*_) and at three temperatures for each sample. The range of temperatures was limited so that the proteins remain native and that the microcrystalline samples would not freeze. In addition to *R*_1ρ_’s, we also measured *R*_1_’s at the same temperatures. *R*_1_’s are not useful for studying rocking motions since these relaxation rates are sensitive to much faster dynamics. At the same time, while fitting the data we need to take into account faster motions as well since without knowledge of the amplitude of the fast motions, the order parameter of the rocking motion cannot be determined precisely.

**Fig. 9:**
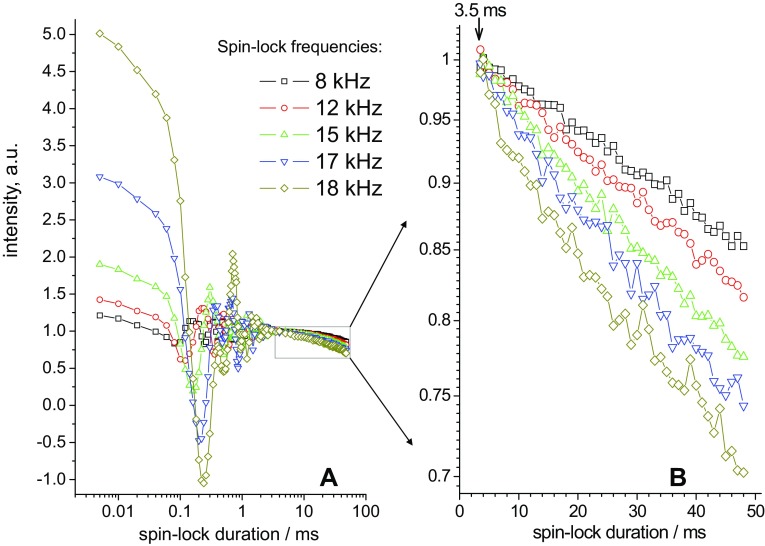
Experimental *R*_1ρ_ decays measured for GB1 at 21.5 °C and 20 kHz MAS for five different spin-lock fields as indicated (**a**). The panel **b** is a zoomed region indicated by the rectangle in the panel **a**. All decays were normalized to unity by the point at 3.5 ms (“dead time”), where the initial oscillations have practically vanished. The experimental error can be assessed from the scatter of the points in the decays

Figure 9 presents typical examples of the *R*_1ρ_ relaxation decays measured at different spin-lock frequencies. Similar decays for one of the ubiquitin samples measured at 40 kHz are shown in ESM, Figs. S1 and S2. Even without any numerical analysis it is clearly seen that the relaxation becomes faster upon approaching spin-lock frequency to the MAS frequency. This is an unambiguous indication of the fact that the protein undergoes dynamics on the microsecond timescale, otherwise the *R*_1ρ_ versus (*ω*_*MAS*_ − *ω*_*SL*_) dependence would be flat (Krushelnitsky et al. 2014; Ma et al. 2014).

**Fig. 10:**
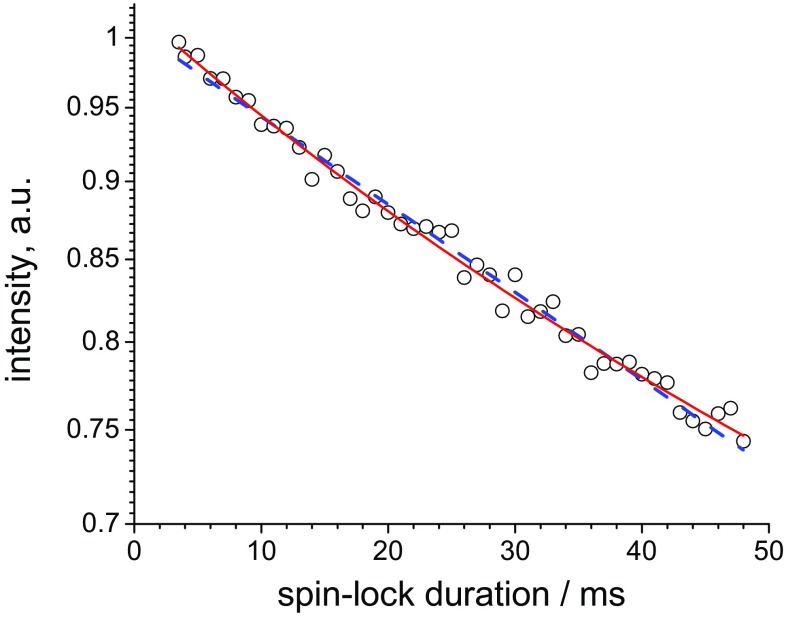
A typical example of the *R*_1ρ_ relaxation decay with barely noticeable multi-exponentiality, measured on GB1 at 21.5 °C, 20 kHz MAS and a spin-lock field of 17 kHz. The dashed blue and solid red lines are single- and bi-exponential fits, respectively. The relaxation rates obtained from the single- and bi-exponential fits are 6.5 ± 0.25 and 8.0 ± 1.0 s^−1^, respectively. In the latter case, the mean relaxation rate (i.e. initial slope) was obtained as $\left\langle {{R_{1\rho }}} \right\rangle =p \cdot {R_{1\rho a}}+(1 - p) \cdot {R_{1\rho b}}$, where *p* and *R*_1ρ*a,b*_ are the relative amplitude of one of the components and relaxation rate constants of two components of the decay, respectively (see Eqs. S1–S4 of ESM). The fitting values of these parameters are: *p* = 0.74 ± 0.5; *R*_1ρ*a*_=2.8 ± 5 s^−1^; *R*_1ρ*b*_=23 ± 20 s^−1^

The initial oscillations were excluded from the analysis and the fitting was performed over all points beyond 3.5 ms spin-lock pulse duration. Figure 10 shows the result of the fitting of a typical relaxation decay using mono- and bi-exponential fitting functions. This is merely a demonstration of the difference between the meaningless mean relaxation time (from the single-exponential fit) and the physically relevant mean relaxation rate (see above) using a typical experimental relaxation decay. The non-exponential shape of the decays is barely seen, tempting one to neglect the non-exponentiality and to fit the decay with a single exponential. However, as it is shown in the figure, the values of the mean time and the mean rate are appreciably different.

After determining the set of the relaxation rates for all samples, one has to fit the data using a specific motional model. We suggest using a model with two motional modes—fast internal conformational motion on the nanosecond timescale and slow overall protein rocking motion. Since we analyse the overall signal from the whole protein, we introduce a distribution of correlation times of the fast internal motion that takes into account the internal dynamic heterogeneity of a protein. Thus, a spectral density function reads

2 $$J(\omega )=(1 - S_{f}^{2})\int\limits_{0}^{\infty } {\rho (\tau ,{\tau _f},\beta )\frac{\tau }{{1+{{(\omega \tau )}^2}}}} d\tau +S_{f}^{2}(1 - S_{S}^{2})\frac{{{\tau _S}}}{{1+{{(\omega {\tau _S})}^2}}}$$

where $S_{{f,S}}^{2}$ and ${\tau _{f,S}}$ are the order parameters and the correlation times of the fast and slow motions, respectively; $\rho (\tau ,{\tau _f},\beta )$ is the distribution function that is parameterized by two parameters—${\tau _f}$, the centre of the distribution and β, the distribution width parameter. Without a distribution of the correlation times for the fast motion, good data fitting was unachievable. As for the distribution of the fast-motion correlation times, we used two phenomenological models, a log-normal and a modified non-symmetric Fuoss-Kirkwood distribution (Schneider 1991), for details see the ESM. The analysis assumes that the order parameters and the shape of the distribution function are the same at all temperatures. While in reality these are obviously temperature-dependent, the temperature dependence is likely rather weak, otherwise the proteins and crystals would not be rigid and stable. Taking the temperature dependence of these parameters by some phenomenological function into account is in principle possible, but this would make the fitting less certain and in any case would not lead to any significant change of the final results.

The slow (rocking) motion likely has a distribution of correlation times that can be caused either by inhomogeneity of a sample (e.g. defects in crystal packing) or an intrinsically complicated shape of the rocking-motion correlation function arising from a possible inter-correlation of motion of neighbouring proteins in a crystal. We tried to fit the data assuming a distribution for the slow motion as well. However, in all cases the inclusion of one more fitting parameter (distribution width for the slow-motion correlation times) leads to a practically negligible improvement of the fitting quality (results not shown). At the same time, the dependence of the main parameters of the slow motion, the correlation time and the order parameter, on the width of the distribution is rather weak. Thus, following the principle of Occam’s razor, we include in the fitting model only a single correlation time of the slow rocking motion.

**Fig. 11:**
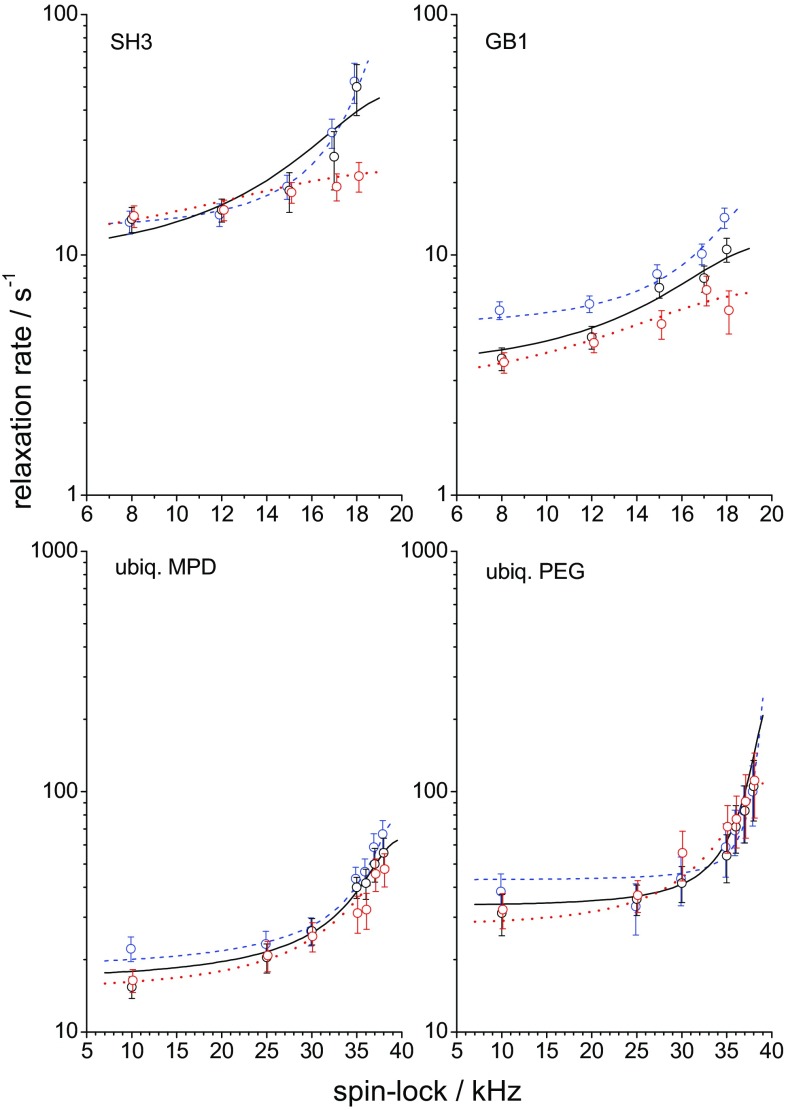
*R*_1ρ_ relaxation rates as a function of spin-lock frequency for four different samples at three different temperatures. Circles—experimental data, solid lines—fitting curves. Blue dashed, black solid and red dotted lines correspond to the temperatures 13, 21.5 and 29 °C for SH3 and GB1 and 3, 15 and 27 °C for two ubiquitin samples, respectively

**Fig. 12:**
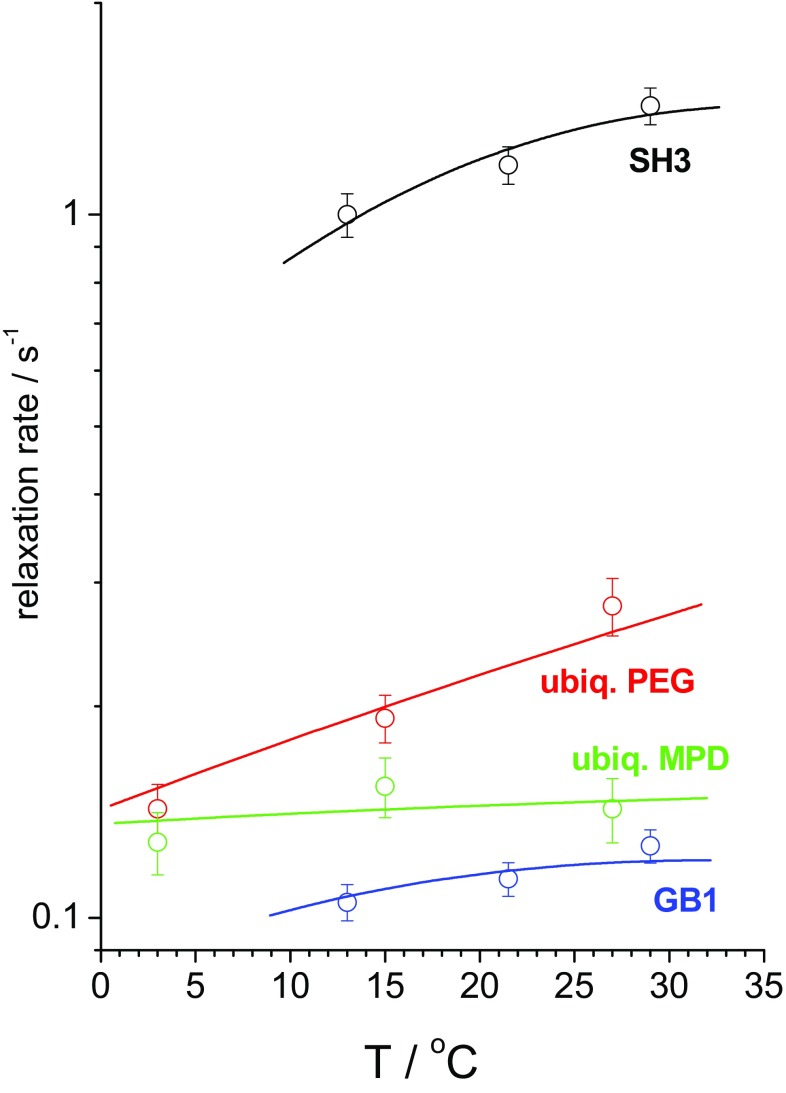
*R*_1_ relaxation rates as a function of temperature for four different samples as marked. Circles—experimental data, solid lines—fitting curves

As expected, the type of the distribution function has an effect on the parameters of the fast motion, but the slow-motion parameters are practically insensitive to it. Since we analyze the relaxation times at different temperatures, we assume an Arrhenius dependence of the correlation times on temperature,

3 $${\tau _{f,S}}=\tau _{{f,S}}^{{20}} \cdot \exp \left( {\frac{{{E_{f,S}}}}{R}\left( {\frac{1}{T} - \frac{1}{{293K}}} \right)} \right)$$

where $\tau _{{f,S}}^{{20}}$ are the correlation times at temperature 20 °C, and ${E_{f,S}}$ are activation energies of the fast and slow motions. Thus, the total number of fit parameters is seven: two order parameters, two correlation times, two activation energies (for the fast and slow motions) and the distribution width parameter for the fast motion. At the same time, the number of experimental points (relaxation rates) was 18–24 for each sample. Further details of the fitting procedure are laid out in the ESM. The parameters of the fast motion may be poorly determined, since only *R*_1_ rate constants at a single resonance frequency contain information on the fast dynamics, which is obviously not enough for a reliable determination of the nanosecond timescale part of the motional correlation function. Also, the activation energies are not determined very precisely since the temperature range of the experiments was quite narrow. Fortunately, the uncertainty of these parameters is a minor problem since the main goal of this work is to assess the timescale of the slow rocking motion and its order parameter, which could be reliably determined from the data since the abundant *R*_1ρ_ rates sample the microsecond range of molecular dynamics in sufficient detail. All the fit parameters for two types of the distribution functions are presented in ESM, see Tables S1 and S2, and below in Table 1 we collect only the most important and relevant results—the order parameters and the correlation times of the slow rocking motion for the four different protein samples. Table 1 also presents the angular amplitudes assuming the motional model to be jumps between two equivalent sites, which gives an impression of the angle of the whole protein reorientation on the microsecond time scale (this of course should not be necessarily the 2-site jumps). The last row in Table 1 is a solvent content in the protein crystals which will be discussed below. Figures 11 and 12 present the experimental relaxation rate constants and the fit curves.

**Table 1:** Order parameters, angular amplitudes (assuming 2-site jumps model) and the correlation times of the rocking motion at 20 °C for four samples

	GB1	SH3	Ubiq. MPD	Ubiq. PEG
$S_{S}^{2}$	0.9995 ± 0.00006	0.9915 ± 0.005	0.9965 ± 0.001	0.987 ± 0.003
Ang.ampl. 2-site jumps	1.5°	6.1°	3.9°	7.6°
$\tau _{S}^{{20}}$/μs	41 ± 5	46 ± 4	30 ± 7	52 ± 7
Solvent content in crystal/%	33.6	49.8	49.6	56

The numbers are averaged values of the fit results obtained using two different models for the τ-distribution functions for the fast motion (see Tables S1 and S2 in ESM for details). The solvent content in the crystals was determined using the molecular weight and unit cell parameters in the PDB entries 2GI9 (GB1), 1U06 (SH3), 3ONS (MPD-ub) and 3N30 (cubic-PEG-ub); the data for all these crystal structures have been collected at 100 K. Note that for a different crystal form of GB1, grown under very similar conditions (MPD, pH 4.5), corresponding to PDB entry 2QMT, the solvent content is also rather low, 45%, with a similar intermolecular β-sheet

The detection of slow motion in all crystals suggests, as mentioned above, the presence of an overall motional process, such as rigid-body rocking (Ma et al. 2015; Lamley et al. 2015b; Kurauskas et al. 2017). Alternatively, the observed very low amplitude of the slow motion in the non-selective experiment could also be explained by an internal conformational motion that affects only a (possibly rather small) fraction of residues. For the case of cubic-PEG-ub, the rigid-body nature of the motion has been confirmed by site-resolved measurements (Ma et al. 2015). For other proteins, especially revealing higher $S_{S}^{2}$, the existence of the whole-body motion is so far not established. A criterion of such overall motion is a common component of the motional correlation function with the same correlation time for all N–H bonds in a protein. To provide evidence for the global character of the motion, we performed site-resolved *R*_1ρ_ experiments in 2D-mode for the GB1 sample, i.e. the sample having the highest $S_{S}^{2}$ out of all four samples studied in the current work. Since collecting the large set of temperatures and RF field strengths that we covered by the 1D experiments would be too time consuming, we performed only two *R*_1ρ_ experiments at two spin-lock frequencies, 8 and 17 kHz, at a single temperature 21.5 °C. Having only two relaxation rate constants does not allow for any reliable quantitative estimation of the order parameters and correlation times, but the difference between the relaxation rates measured at two spin-lock fields unambiguously reveals the presence of μs motion. If the motion is faster or slower in comparison to a 10^−6^–10^−4^ s correlation time range, then the difference is zero, otherwise the difference has an appreciable value proportional to the amplitude of the microsecond motion.

**Fig. 13:**
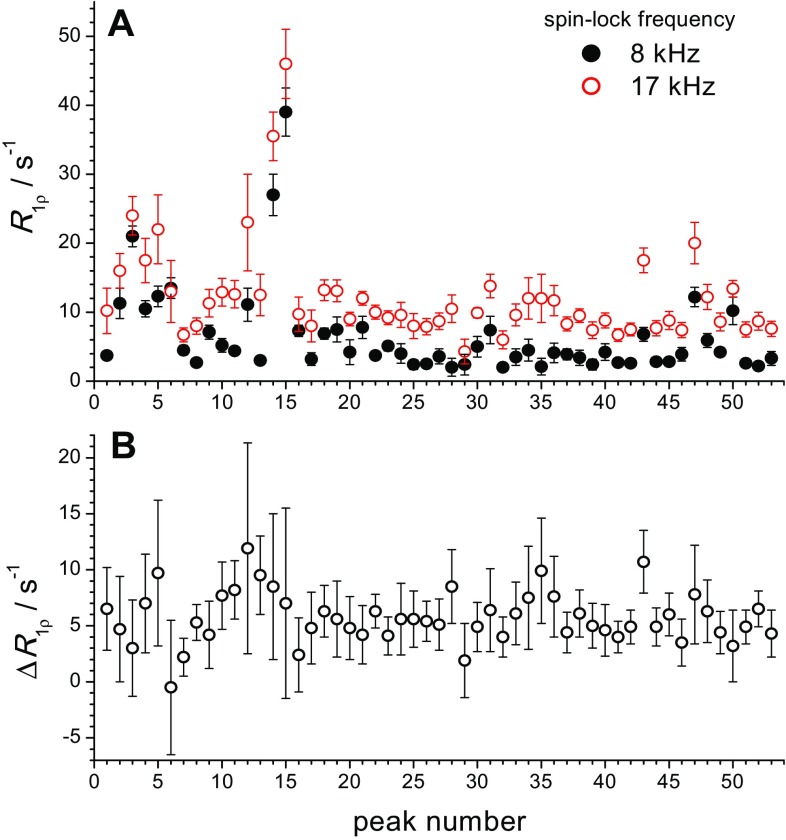
Relaxation rates for different peaks in 2D-spectrum measured in GB1 at 21.5 °C and MAS 20 kHz with spin-lock frequencies of 8 and 17 kHz (**a**), and difference *R*_1ρ_(17 kHz)–*R*_1ρ_(8 kHz) (**b**), both plotted vs the peak number (which does not correspond to the primary structure of the protein)

Figure 13 presents the relaxation rates *R*_1ρ_ measured at two spin-lock fields as well as their difference. In this analysis, we have only used arbitrary peak numbers, rather than residue numbers. The spectrum of our sample shown in Fig. S3 (ESM), obtained from similar crystallization conditions as previously reported ones (see “Materials and methods” section) differs from the previously published spectra to a point where unambiguous assignment of peaks to residues is possible only for 2–3 peaks. For the present analysis, the assignment is not crucial, in particular as we find indeed a very similar behaviour for all residues.

Except for one peak (labelled as peak no. 6), all residues reveal a positive difference, *R*_1ρ_(17 kHz)—*R*_1ρ_(8 kHz), which indicates that all parts of the protein undergo the same type of motion on the μs timescale. Although the error bars in Fig. 13b are rather large, the decays themselves, even without the analysis, leave no doubt about the uniform *R*_1ρ_ spin-lock frequency dependence, see Fig. S4 (ESM). The absolute value of this difference for the majority of peaks is very similar, around 5 ± 2 s^−1^, which is in good agreement with the corresponding value from the 1D relaxation experiment, 4.3 s^−1^ (Fig. 11). This finding fits much better to the overall protein rocking motion rather than internal conformational dynamics since in the latter case only a part of residues would reveal such a dependence of *R*_1ρ_ on the frequency difference *ω*_*MAS *_ −  *ω*_*SL*_.

Several residues in the protein have elevated relaxation rates in comparison to the mean level, e.g. those corresponding to the peak numbers 3, 4, 5,12, 14, 15, 43, 47 and few others (Fig. 13a). These residues obviously undergo μs time scale motion and this motion is attributed to the internal conformational dynamics since only a small portion of residues reveal such a behaviour. This motion for most peaks is however much faster (the correlation time presumably around 1 μs or shorter) than the overall rocking motion observed in the 1D experiments because the *R*_1ρ_ difference (Fig. 13b) reveals no correlation with the absolute values of the relaxation rates. It follows from the fact that *R*_1ρ_ versus (*ω*_*MAS −*_ *ω*_*SL*_) dependence for the motions with a correlation time around 1 μs and shorter is flat, see Fig. 1 in (Krushelnitsky et al. 2014). Thus, this internal motion does not contribute to the rocking motion parameters obtained from the 1D data. The only exception is the peak no. 43: for this signal, the elevated rates reveal also the elevated difference between the rates, which indicates an additional internal motion with a correlation time of the order of (*ω*_*MAS *_ −  *ω*_*SL*_)^−1^.

We need to admit that the available 2D data do not prove that there are no additional internal conformational motion with a correlation time around 30–50 μs, i.e. the motion that undergoes only a part of residues in the protein. The accuracy of these data is rather low and probably this hypothetical motion is “hidden” within the error bars in Fig. 13. If this is true, the rocking motion amplitude has a contribution from this internal motion. On the other hand, the 2D data demonstrate that all the protein structure is dynamic on the μs timescale, and that the level of this mobility is roughly the same across the different parts of the protein. At the same time, the presence of the overall rocking motion does not necessarily mean that the protein itself remains fully rigid on the μs timescale. The rocking motion is possibly associated with a bend or a twist of the whole structure, and thus discriminating between the whole protein reorientation and global structural plasticity is not a simple problem. Thus, the term “rocking motion” in some cases may imply rather some global dynamics than the restricted rigid body rotation only. Based on the analysis of MD simulations and NMR data, it has been suggested that local microsecond motion may be coupled to overall rocking motion (Kurauskas et al. 2017). Specifically, the reorientation of molecules within the crystal lattice is accompanied by the breaking and formation of a different set of inter-molecular interactions, which likely influence also the interconversion of different internal local conformations. Nonetheless, while we cannot exclude the presence of additional local motions having a correlation time close to that of global protein rocking, the uniformity of the *R*_1ρ_(17 kHz)–*R*_1ρ_(8 kHz) difference strongly suggests an overall/global motion, even for an order parameter as high as 0.9995.

As a further support for the global character of the detected motion, the two ubiquitin samples used in this study have previously been studied in a site-resolved manner, and, similarly to the case of GB1, a RF-field dependence of *R*_1ρ_ has been observed over all residues in the cubic-PEG-ub sample (Kurauskas et al. 2017). A global fit of the site-specific near-rotary-resonance *R*_1ρ_ relaxation dispersion profiles of 22 available well-resolved amide sites in cubic-PEG-ub had been performed, and the obtained motional time constant was in the range of tens of microseconds, which is in excellent agreement with the value found in the present study. The motional amplitude had previously been estimated to be ca. 3°–5°, corresponding to an order parameter ca. 0.98. Independent MD simulations had estimated an order parameter of overall rocking motion of ca 0.95–0.98. These values are also in a satisfactory agreement with the value found here, S^2^ = 0.987.

The availability data of four different protein crystals provides interesting insight into the nature of rocking motion and allows us to suggest an explanation why the rocking motion amplitudes in these samples are so different, varying by a factor of 5 when expressed as an effective rocking-motion angle. The solvent content of these crystals varies from 33% (GB1) to 56% (cubic-PEG-ub), see Table 1, with a good qualitative correlation with the amplitude of the rocking motion. For the case of GB1, which shows the lowest-amplitude overall motion, it is interesting to note that the proteins form inter-molecular β-sheets, extending through the entire crystal, with three NH···O=C hydrogen bonds connecting the outermost β-strands of neighbouring molecules, see Fig. S5 (ESM). This tight interaction and packing of neighbouring molecules obviously limits the slow overall motion, providing a plausible explanation for the observed low amplitude.

While the overall-rocking motion amplitude differs significantly between the different protein crystals, the previously determined fast local motional amplitudes of NH bonds appear to be more similar for GB1 (Mollica et al. 2012), SH3 (Chevelkov et al. 2009) and ubiquitin (Haller and Schanda 2013; Ma et al. 2015). The observation that the local motion is less influenced by crystal packing is not surprising as it has been established that the internal motion amplitudes in proteins are dictated by the local density of the internal protein structure (Halle 2002). Local structural density in a protein and packing density of protein globules in a crystal of course should not be necessarily correlated, however, in both cases they seem to be a crucial factor affecting the amplitude of the fast internal and slow overall motions, respectively.

Despite the very different rocking amplitudes, the time scales for all samples are rather uniform from ca. 30 to 50 µs, without correlation to the amplitude. While we can only speculate about the origins of this similarity of time scales, this observation may indicate that the rocking motion is not a diffusive but rather a jump-like process. For a restricted diffusive motional model, e.g. wobbling in a cone or rotational diffusion around an axis, the apparent correlation time would be dependent on the amplitude of motion, assuming that the diffusion coefficient is the same. Such a dependence has been observed e.g. in numerical simulations of the motional correlation function in the Supporting Information to ref. (Zinkevich et al. 2013). For jumps between a small number of sites, the correlation time does not depend on the amplitude. Thus, the rocking motion is likely a jump-like process between different molecular orientations. These different orientations or protein molecules in the crystal certainly differ in the pattern of inter-molecular interactions. Indeed, long MD simulation of different ubiquitin crystals with up to 48 molecules have revealed a pattern of fluctuating intermolecular salt bridges and hydrogen bonds, and have suggested that different orientational states of the protein are stabilized each by a different set of interactions (Kurauskas et al. 2017). Thus, each set of interactions defines a certain energy minimum (a certain protein orientation). However, we are not yet sure that the life time of these inter-molecular interactions and hence, the overall-motion time scale is a universal value for all protein crystals, this obviously requires further experimental evidences.

Lastly, we note that the very high order parameter of the rocking motion can be not only the result of a small angular amplitude between similarly populated states, but could also be due to skewed populations of states which have a larger difference in their relative orientations. Previously observed high order parameters of the microsecond motion in SH3 were ascribed to a concept of low-populated (excited) states that implies jumps between conformational states with non-equal probabilities (Zinkevich et al. 2013). We now think that this microsecond motion is not internal but overall rocking motion, still the latter can be associated with the jumps between non-equal probability states. However, the available data do not allow making definite conclusions on this issue.

## Conclusions

In this work we explored the capabilities and practical limitations of studying slow molecular motions by means of ^15^N *R*_1ρ_ experiments in the vicinity of the rotary resonance conditions. A small difference between the spin-lock and MAS frequencies allows expanding the frequency range of the sampled molecular motions towards rather low frequencies. At the same time, practical problems should be properly handled while conducting the experiments. The most important methodological issues are as follows.

- Upon approaching to the rotary resonance condition, the amplitude of the useful relaxation signal decreases significantly. Consequently, one should seek a compromise between a small difference between spin-lock and MAS frequencies (if the slow dynamics is the target of study) and acceptable signal to noise ratio. When performing a series of measurements at different spin-lock RF field strengths, the measurement time may be distributed such that the sensitivity drop at near-rotary-resonance conditions is compensated. If the signal is very strong and sensitivity is not a limiting factor, then one should keep the *ω*_*MAS*_ − *ω*_*SL*_ difference at least somewhat larger than the width of *ν*_*SL*_-distribution due to *B*_1_-field inhomogeneity. Otherwise, the quantitative analysis of the relaxation data would be too uncertain.
- During the first few milliseconds of the *R*_1ρ_ decay, the useful relaxation signal is subject to initial coherent oscillations. The proper analysis of these oscillations is complex and difficult, and we suggest to consider this initial part of the decay as an effective “dead time” and not to include it in the analysis.
- One should avoid not only rotary resonance but also HORROR (*ω*_*SL*_ = *ω*_*MAS*_/2) conditions. Homonuclear ^15^N–^15^N interaction in proteins is weak, but it is still capable of distorting the relaxation decays significantly. This distortion is even more difficult to handle because of its low frequency, thus precluding simple time averaging; it thus affects the apparent initial decay rate the is the most relevant quantity.
- *R*_1_ and *R*_1ρ_ relaxation decays in solids are always non-exponential. Quite often this non-exponentiality is hardly seen in experiments, still, the decays are non-exponential. For correct quantitative analysis of the relaxation data in terms of the correlation function formalism, one needs to determine not the mean relaxation time but the mean relaxation rate, the latter being the initial slope of the relaxation decay.

Making use of the fact that the 1D mode of the relaxation experiments allows much faster measurement, multiple ^15^N *R*_1ρ_’s at different spin-lock frequencies and temperatures were measured for four different microcrystalline protein samples in order to determine the parameters of the overall rocking motion. The results show that the correlation time of the rocking motion is almost the same for all samples (30–50 μs), however, the amplitudes are very different. The absolute values of the order parameter of the rocking motion are rather high; the most rigid protein, GB1, has an order parameter of 0.9995. The *R*_1ρ_ experiments at two different spin-lock fields performed for this sample in 2D mode show that the very small amplitude corresponds to the overall motion of the whole protein, not internal conformational dynamics of only a (possibly small) part of the protein structure. Thus, the rocking motion seems to be a general feature of proteins in a crystal. The rocking motion amplitude of the four samples correlates with the solvent content in the protein crystal suggesting that the amplitude depends on the packing density of a protein crystal. There are indications that the rocking motion is likely a jump-like dynamic process, this however should be confirmed in future studies.

## Electronic supplementary material

Below is the link to the electronic supplementary material.

Supplementary material 1 (PDF 487 KB)
