# Supplementary material for: Microsecond motions probed by near-rotary-resonance R1ρ15N MAS NMR experiments: the model case of protein overall-rocking in crystals
Source: J Biomol NMR. 2018 May 30;71(1):53–67. doi: 10.1007/s10858-018-0191-4 (PMC5986846; doi:10.1007/s10858-018-0191-4)
Supplement: Supplementary file 1 — Supplementary material 1 (PDF 487 KB) [file 10858_2018_191_MOESM1_ESM.pdf]

Electronic Supporting Material

Microsecond motions probed by near-rotary-resonance $R_{1\rho}$ ^{15}N MAS NMR experiments – the model case of protein overall-rocking in crystals

Alexey Krushelnitsky[1]*, Diego Gauto[2], Diana C. Rodriguez Camargo[3], Paul Schanda[2], Kay Saalwächter[1]

[1] Martin-Luther-Universität Halle-Wittenberg, Halle, Germany;

[2] Institut de Biologie Structurale (IBS), Grenoble Cedex 9, France;

[3] Technische Universität München, Garching, Germany (current affiliation: Wren Therapeutics Ltd., Cambridge, United Kingdom)

Data analysis

Analysis of non-exponential decays

Here we present some practical hints that can be useful for the analysis of the experimental (in general case non-exponential) relaxation decays. As described in the main text, the simplest fitting function for such decays is a sum of two exponentials:

$$A(t) = A_0 \cdot \left[p \cdot \exp(-R_{1\rho a} \cdot t) + (1-p) \cdot \exp(-R_{1\rho b} \cdot t) \right], \qquad \text{(S1)}$$

where A_0, p, $R_{1\rho a}$ and $R_{1\rho b}$ are the fitting parameters. The parameters p, $R_{1\rho a}$ and $R_{1\rho b}$ themselves have no physical meaning, however out of them the mean relaxation rate can be easily calculated:

$$\left\langle R_{1\rho} \right\rangle = p \cdot R_{1\rho a} + (1-p) \cdot R_{1\rho b} . \qquad \text{(S2)}$$

In practice it more convenient to express $R_{1\rho b}$ as a function of $R_{1\rho a}$ and $\left\langle R_{1\rho} \right\rangle$ using Eq. (S2)

$$R_{1\rho b} = \left(\left\langle R_{1\rho} \right\rangle - p \cdot R_{1\rho a} \right) / (1-p) \qquad \text{(S3)}$$

and then insert this expression in Eq. (S1):

$$A(t) = A_0 \cdot \left[p \cdot \exp(-R_{1\rho a} \cdot t) + (1-p) \cdot \exp\left(-t \cdot \left(\langle R_{1\rho} \rangle - p \cdot R_{1\rho a} \right) / (1-p) \right) \right]. \quad \text{(S4)}$$

This form of the fitting function has the fitting parameters A_0, p, $R_{1\rho a}$ and $\langle R_{1\rho} \rangle$. Thus, $\langle R_{1\rho} \rangle$ can be obtained from the fitting directly without additional calculation using Eq. (2). It is also important that the fitting uncertainty for $\langle R_{1\rho} \rangle$ in this case can also be obtained from the fitting software directly without additional calculations. One should be aware of the fact that the fitting uncertainties of p, $R_{1\rho a}$ and $R_{1\rho b}$ are usually rather high. However, the uncertainty of $\langle R_{1\rho} \rangle$ is much smaller since the parameters p, $R_{1\rho a}$ and $R_{1\rho b}$ are highly inter-correlated. This is illustrated in Fig. 10 of the main paper. Few other hints are as follows:

- if the relaxation decay is too curved and double-exponent (or a phenomenological distribution of the relaxation times) fitting function is not sufficient for a good fitting, then the fitting range of the decay can be shortened, i.e. one should fit not the whole decay, but its initial part, where good fitting is achievable. Alternatively, the number of components in the fitting function can be increased, this is, however, a more complex and less definite approach;
- one should avoid the very small relative amplitude (around few percent) of the fast component while using the double-exponential fitting function. In most cases such a small amplitude means that a computer tries to fit the experimental noise at the very beginning of the decay. This can be achieved by limiting the allowed values of the relative amplitude during the fitting. For the same reason it is not advisable to use excessive number of components in the fitting function since the fitting software again would try to fit noise at the beginning of the relaxation decay assuming a relaxation component with a very large (sometimes even negative) relaxation rate. This may significantly distort the result;
- if the signal-to-noise ratio is not sufficient or if the length of a decay is not long enough so that the multi-exponential shape of the decay is not seen, then fitting the decay using single- and double-exponential (or distribution) fitting function provides practically the same mean relaxation rate and the same fitting error. In this case, applying more complex fitting function of course does not make sense.

Finally, we note that in general, the fitting uncertainty of the mean relaxation rate constant obtained using double-exponential (or distribution) fitting function is appreciably larger than the uncertainty

obtained using the single-exponential fit. This however cannot be an argument for using the simplest fitting function except the case considered in the preceding paragraph.

Relaxation rate constants

The experimentally measured ^{15}N relaxation rate constant $R_{1\rho}$ is a sum of two terms,

$$R_{1\rho} = R_{1\rho}^{NH} + R_{1\rho}^{CSA}, \tag{S5}$$

where $R_{1\rho}^{NH}$ and $R_{1\rho}^{CSA}$ are the rate constants corresponding to the heteronuclear dipolar and chemical shift anisotropy relaxation mechanisms, respectively. The two rate constants can be expressed as a function of the motional spectral density function as follows:

$$\begin{aligned} R_{1\rho}^{NH} = \frac{K_{NH}^2}{20}\Big[& \big(2J(\omega_{SL} - 2\omega_{MAS}) + 4J(\omega_{SL} - \omega_{MAS}) + 4J(\omega_{SL} + \omega_{MAS}) + 2J(\omega_{SL} + 2\omega_{MAS})\big)/3 + \\ & + J(\omega_N - \omega_H) + 3J(\omega_N) + 6J(\omega_H) + 6J(\omega_N + \omega_H)\Big] \end{aligned} \tag{S6}$$

$$\begin{aligned} R_{1\rho}^{CSA} = \frac{(\delta\omega_N)^2}{135} & \big(2J(\omega_{SL} - 2\omega_{MAS}) + 4J(\omega_{SL} - \omega_{MAS}) + \\ & + 4J(\omega_{SL} + \omega_{MAS}) + 2J(\omega_{SL} + 2\omega_{MAS}) + 9J(\omega_N)\big) \end{aligned}, \tag{S7}$$

where $\omega_{SL}/2\pi$ and $\omega_{MAS}/2\pi$ are the spin-lock and MAS frequencies, $\omega_N/2\pi$ and $\omega_H/2\pi$ are the ^{15}N and ^{1}H resonance frequencies, K_{NH}^2 is the square of the powder-averaged ^{15}N-^{1}H dipolar interaction and δ is the CSA interaction strength, i.e. the difference between the parallel and perpendicular components of the axially symmetric CSA tensor. In the analysis, we assumed for K_{NH}^2 and δ the standard values for protein backbone nitrogens of $5.2 \cdot 10^9$ s^{-2} and 160 ppm, respectively.

Note that Eqs. (S6) and (S7) are valid only for the on-resonance spin-lock experiment; if the spin-lock pulse is applied with a non-negligible resonance offset, then general formulae should be used, see details in (Kurbanov et al. 2011; Krushelnitsky et al. 2014). Eq. (S6) corresponds to the case of heteronuclear dipolar relaxation mechanism, homonuclear relaxation under MAS should be analyzed using equations described in (Rovo et al. 2017).

The corresponding formulae for R_1 are:

$$R_1^{NH} = \frac{K_{NH}^2}{10}\left(J(\omega_N - \omega_H) + 3J(\omega_N) + 6J(\omega_N + \omega_H)\right) \tag{S8}$$

$$R_1^{CSA} = \frac{2}{15}\left(\delta\omega_N\right)^2 J(\omega_N). \tag{S9}$$

The above treatment neglects the effects of dipolar-CSA cross-correlated relaxation (CCR), which would lead to differential relaxation of the different ^{15}N double components. Experimentally the effects of cross-correlated relaxation have been suppressed by applying a proton π-pulse in the middle of the ^{15}N-spin-lock pulse, as mentioned in the experimental section of the main text, and, thus, we can safely neglect the CCR effects.

Spectral density function

As explained in the main text, the spectral density function was presented as a sum of two terms corresponding to the fast and slow motions, the former with a distribution of the correlation times:

$$J(\omega) = (1 - S_f^2)\int_0^\infty \rho(\tau, \tau_f, \beta)\frac{\tau}{1 + (\omega\tau)^2}d\tau + S_f^2(1 - S_S^2)\frac{\tau_S}{1 + (\omega\tau_S)^2}. \tag{S10}$$

In the analysis, we used two kinds of phenomenological distribution functions. The first is the modified Fuoss-Kirkwood distribution function (Schneider 1991). Assuming this type of distribution, the whole distribution integral over the τ-dependent Lorentzian spectral density in Eq. (S10) reads:

$$J(\omega) = \frac{\alpha + \beta}{2}\cdot\sin\left(\frac{\alpha\pi}{\alpha + \beta}\right)\cdot\frac{1}{\omega}\cdot\frac{\left(\omega\tau_f\right)^\alpha}{\left(1 + \left(\omega\tau_f\right)^{\alpha+\beta}\right)}. \tag{S11}$$

Here, τ_f is the mid-distribution correlation time, α and β are the distribution width parameters (0<α<1, 0<β<1) that regulate the slow (long correlation times) and fast (short correlation times) wings of the distribution function, respectively. If α=β=1, the distribution is infinitely narrow (i.e., a δ-function), if α=β=0, the distribution is infinitely wide. The case α=β corresponds to the well-known Fuoss-Kirkwood distribution function, and if α≠β, then the distribution becomes non-symmetric. In our analysis we always assume non-symmetric distribution, α was fixed at 1 and β was a variable fitting parameter. We did so since this distribution has very slowly decaying wings, and if α<1, the part of the fast motion distribution will always overlap the slow (rocking motion). This would lead to the incorrect order parameter of the slow motion obtained from the fitting. The

non-symmetric shape of the correlation time distribution for the fast motion is of course unphysical and it may provide biased information on the fast motion parameters. This is however not critical for the present study since we are not interested in precise and correct fast-motion parameters, we just need to take it into account formally, to obtain correct values of the rocking motion parameters.

The second type of the distribution function we tested is the log-normal distribution,

$$\rho(\tau,\tau_f,\beta)=\frac{1}{\tau\beta\sqrt{2\pi}}\exp\left[\frac{-(\ln[\tau]-\ln[\tau_f])^2}{2\beta^2}\right], \tag{S12}$$

where β is also a distribution width parameter (0<β<∞); the larger β, the wider the distribution. In this case, the integral in Eq.(S10) was calculated numerically during the fitting procedure. The temperature dependence of the correlation times was assumed to be Arrhenian, see Eq.(3) of the main paper.

Fitting results

The whole set of relaxation data for each sample can be described by a following set of fitting parameters:

$S^2_{f,S}$ - order parameters of the fast and slow motions;

$\tau^{20}_{f,S}$ - correlation times of the fast and slow motions at 20 ºC;

$E_{f,S}$ - activation energies of the fast and slow motions;

β - distribution width parameter for the fast motion (two different distributions, see above).

The data fitting was performed using a Monte-Carlo algorithm aiming to minimize the root-mean-square deviation

$$RMSD=\sqrt{\frac{1}{N}\sum_{i=1}^{N}\left(\frac{R^i_{\exp}-R^i_{sim}}{R^i_{\exp}}\right)^2}, \tag{9}$$

where N is the number of experimental points (including all $R_{1\rho}$ and R_1 rate constants), $R^i_{\exp}$ and R^i_{sim} are the experimental and simulated $R_{1\rho}$ or R_1 relaxation rate constants, respectively, according to the formalism described above. From the Monte-Carlo trajectories (each trajectory includes from 10^5 to 10^6 accepted steps), the mean values as well as rmsd values for each fitting parameter were determined. Tables 1 and 2 below present the fitting results assuming the modified Fuoss-Kirkwood (Tab. S1) and log-normal (Tab. S2) distributions for the fast motion.

Table S1. Fitting results assuming the modified Fuoss-Kirkwood distribution of the fast-motion correlation times. The fitting parameters errors were determined from the MC trajectories and thus present only the fitting uncertainty.

	Fitting parameters	GB1	SH3	Ubiq. MPD	Ubiq. PEG
Fast motion	S_f^2	0.867±0.02	0.2±0.05	0.78±0.03	0.39±0.05
	τ_f^{20} / ns	74±10	13±2	140±80	47±8
	E_f / kJ/mol	50±10	54±6	11.2±1.5	6±3
	β	0.18±0.06	0.41±0.08	0.38±0.16	0.94±0.08
Slow motion	S_S^2	0.99945±0.00006	0.989±0.003	0.9961±0.0003	0.987±0.002
	τ_S^{20} / µs	42±3	47±3	28±2	53±6
	E_S / kJ/mol	55±8	107±8	18±3	31.5±10
RMSD		0.07	0.12	0.07	0.12

Table S2. Fitting results assuming the log-normal distribution of the fast-motion correlation times.

	Fitting parameters	GB1	SH3	Ubiq. MPD	Ubiq. PEG
Fast motion	S_f^2	0.92±0.02	0.34±0.02	0.87±0.01	0.41±0.07
	τ_f^{20} / ns	6.5±1.5	6±1	10±5	42±2
	E_f / kJ/mol	44±5	41±4	15±2	7±3
	β	1.85±0.2	0.95±0.2	2.5±0.5	0.36±0.25
Slow motion	S_S^2	0.9995±0.00005	0.994±0.0005	0.9969±0.0005	0.988±0.002
	τ_S^{20} / μs	40.5±4	46±3	31±5	52±5
	E_S / kJ/mol	46±5	95±6	19±4	33±10
RMSD		0.07	0.12	0.07	0.12

We would like to note that relatively low order parameters of the fast motion, especially for the SH3 and ubiquitin PEG samples, are not an indication of the high average amplitude of the backbone motion. The integral signal also includes side-chain nitrogens that undergo fast motion with a high amplitude. The contribution of the side chain nitrogens to the integral signal is specific for a sample and the CP conditions. For this reason, S_f^2 contains practically no physically relevant information.

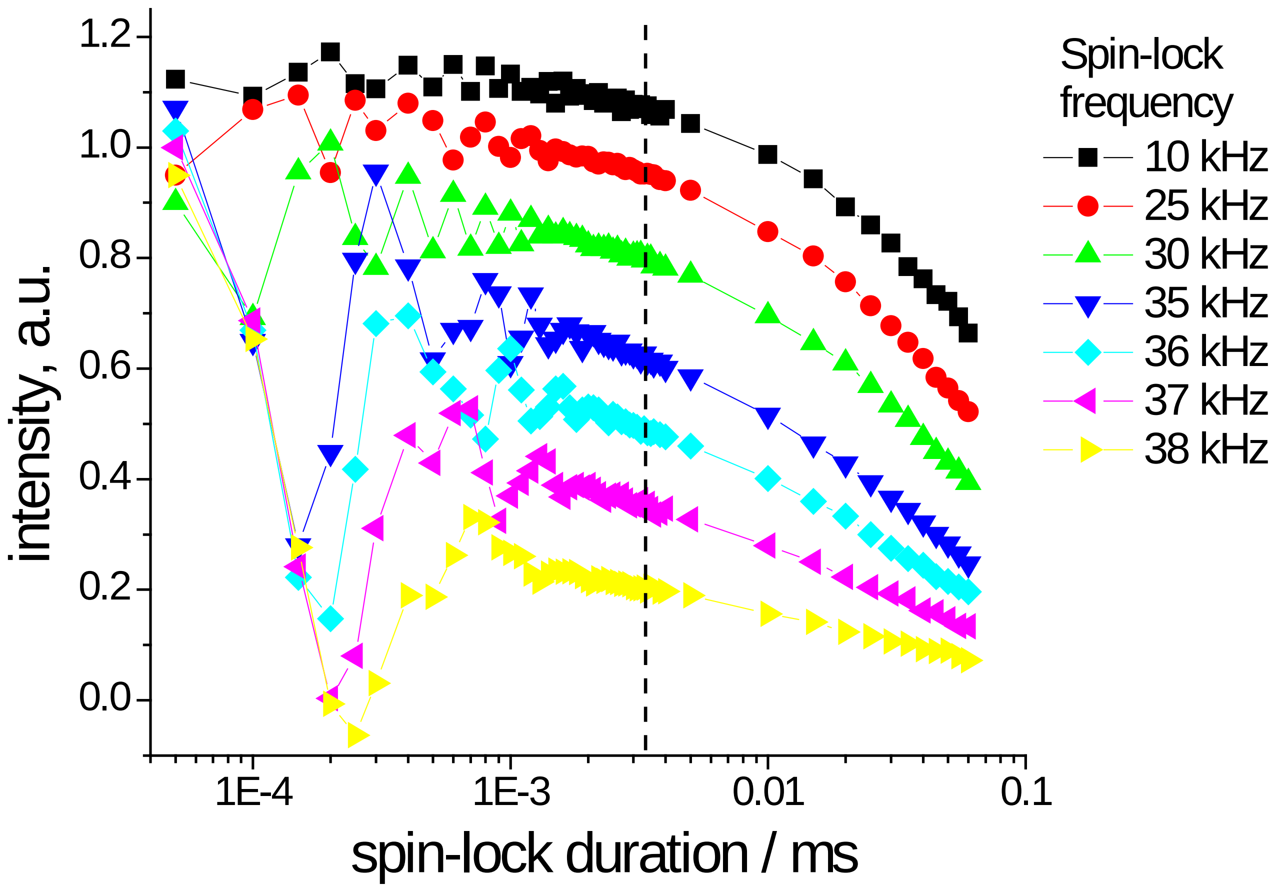

Figure S1. $R_{1\rho}$-decays of MPD-ubiquitin sample at 15 °C and different spin-lock frequencies (indicated in the figure). The decays were arbitrarily normalized for better viewing. The dashed line indicates the time point, at which the initial oscillation are vanished. The experimental points at shorter delays in respect to this point were not taken into the analysis.

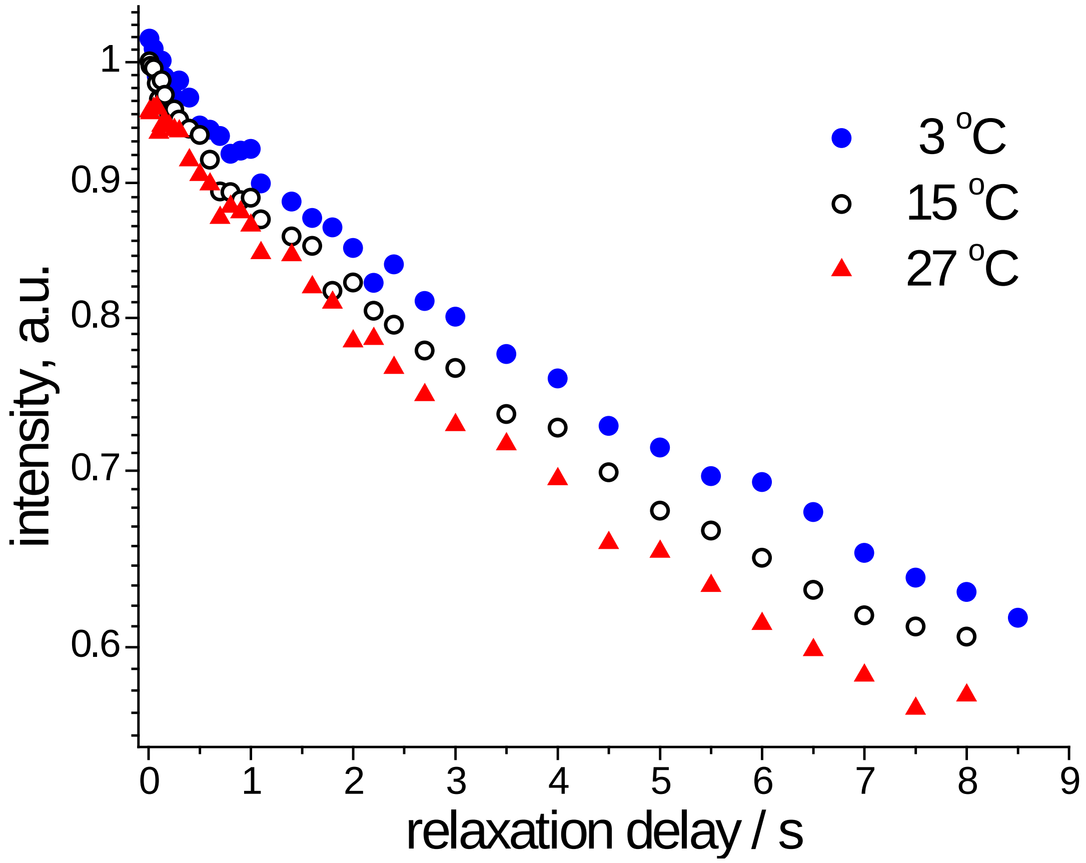

Figure S2. R_1-decays of MPD-ubiquitin sample measured at three temperatures (indicated in the figure).

2D relaxation data for GB1 sample

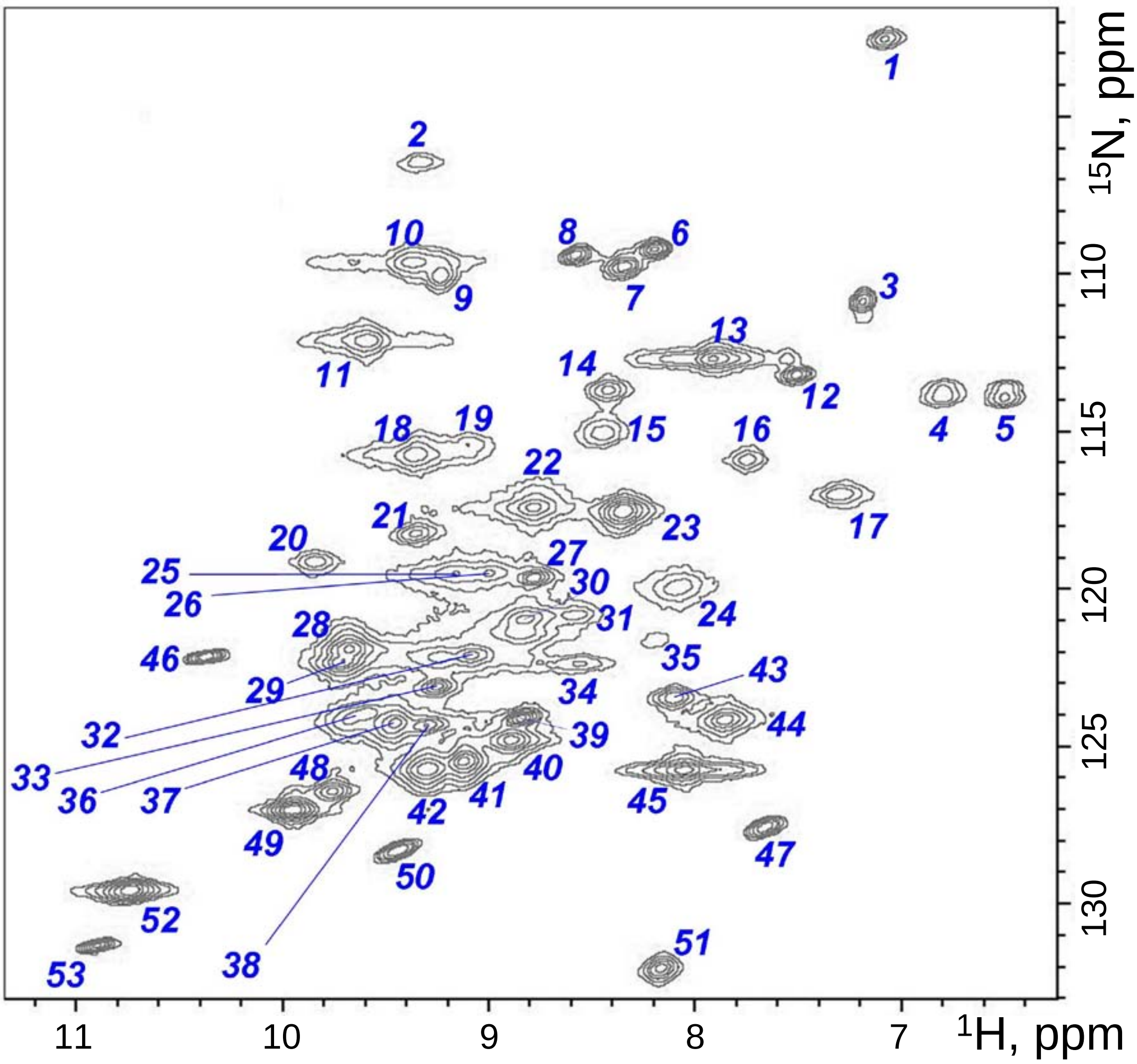

Figure S3. ^{1}H-^{15}N correlation spectrum of the GB1 sample with the numbering of the peaks used in Fig. 13 of the main paper. The difference between the pattern of this 2D spectrum and that of the GB1 spectra published previously (Zhou et al. 2007; Lamley et al. 2015) is caused most probably by a usage of a mixture of deuterated (80%) and protonated (20%) solvents instead of natural abundance ones, resulting in one of possible polymorphs of the GB1 microcrystals (Schmidt et al. 2007). The assignment experiments are not feasible since the available to us sample is not ^{13}C-enriched. Note that judging from the line resolution in the 2D spectrum and from the fact that GB1 reveals the smallest relaxation rates R_1 and $R_{1\rho}$ out of all four samples, this protein certainly has a well-defined crystal structure and is not somehow degraded.

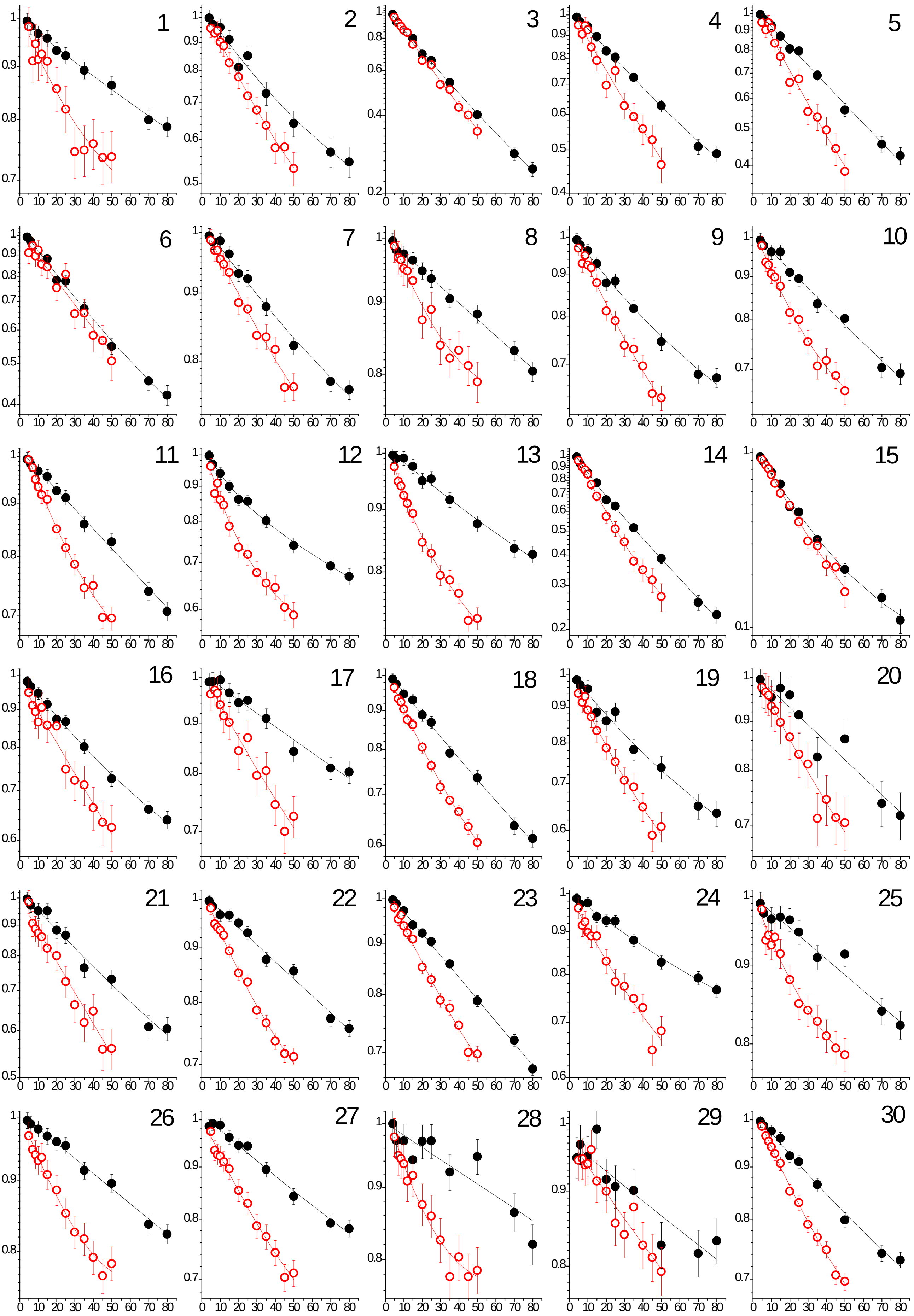
1
1 0.9 0.8 0.7
2
1 0.9 0.8 0.7 0.6 0.5
3
1 0.8 0.6 0.4 0.2
4
1 0.9 0.8 0.7 0.6 0.5 0.4
5
1 0.9 0.8 0.7 0.6 0.5 0.4
6
1 0.9 0.8 0.7 0.6 0.5 0.4
7
1 0.9 0.8
8
1 0.9 0.8
9
1 0.9 0.8 0.7
10
1 0.9 0.8 0.7
11
1 0.9 0.8 0.7
12
1 0.9 0.8 0.7 0.6
13
1 0.9 0.8
14
1 0.9 0.8 0.7 0.6 0.5 0.4 0.3 0.2
15
1 0.1
16
1 0.9 0.8 0.7 0.6
17
1 0.9 0.8 0.7
18
1 0.9 0.8 0.7 0.6
19
1 0.9 0.8 0.7 0.6
20
1 0.9 0.8 0.7
21
1 0.9 0.8 0.7 0.6 0.5
22
1 0.9 0.8 0.7
23
1 0.9 0.8 0.7
24
1 0.9 0.8 0.7 0.6
25
1 0.9 0.8
26
1 0.9 0.8
27
1 0.9 0.8 0.7
28
1 0.9 0.8
29
1 0.9 0.8
30
1 0.9 0.8 0.7
0 10 20 30 40 50 60 70 80

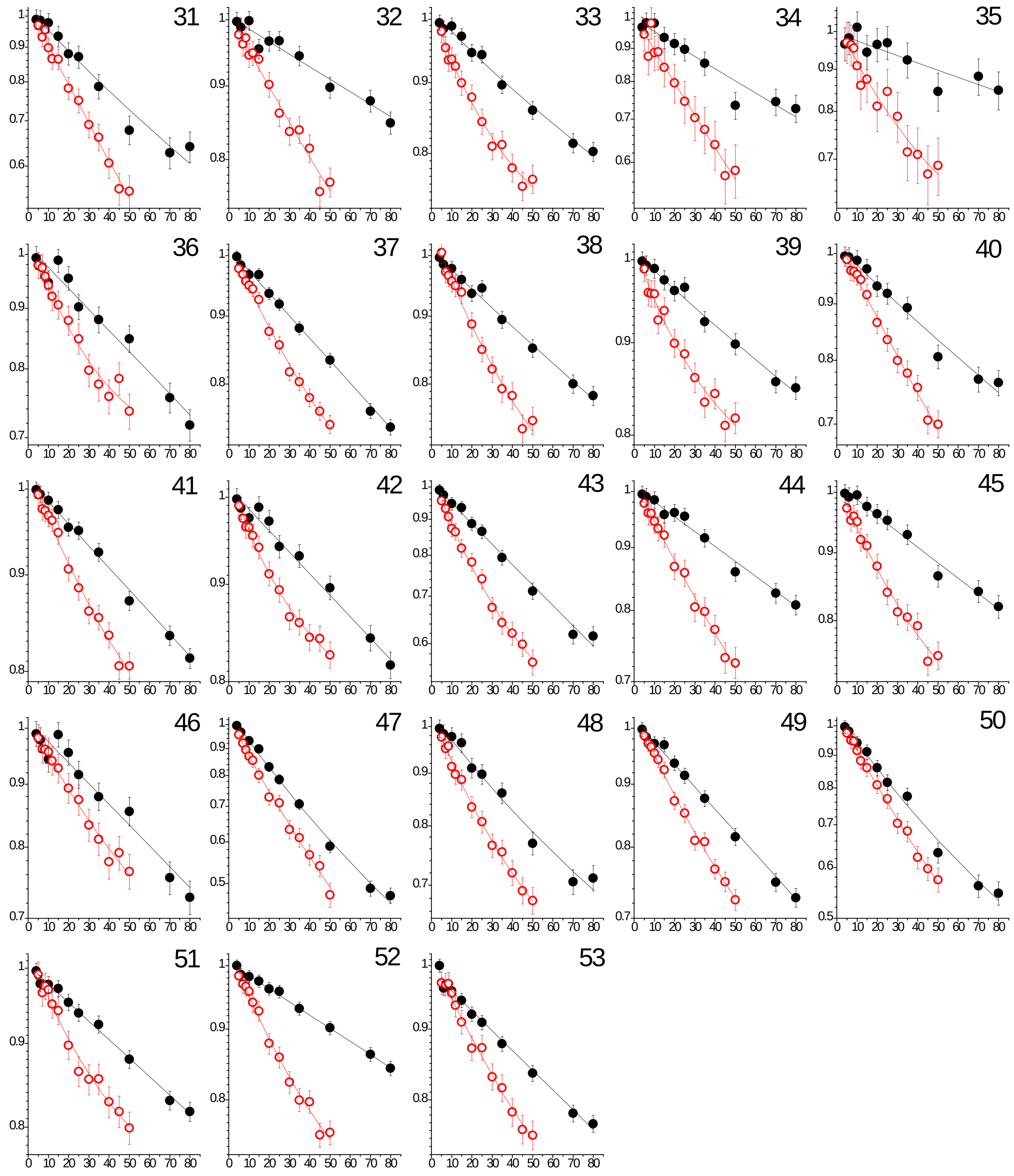

Figure S4. $R_{1\rho}$ relaxation decays for 53 peaks in the 2D spectrum of GB1 sample (Fig. S3) measured at spin-lock frequencies 8 kHz (black solid circles) and 17 kHz (red open circles). The numbering of the plots corresponds to the numbering of the peaks in Fig. S3. Solid lines are the fitting curves, the fitting was performed according to the hints described in the first part of ESM. All x-axes correspond to the spin-lock duration in ms.

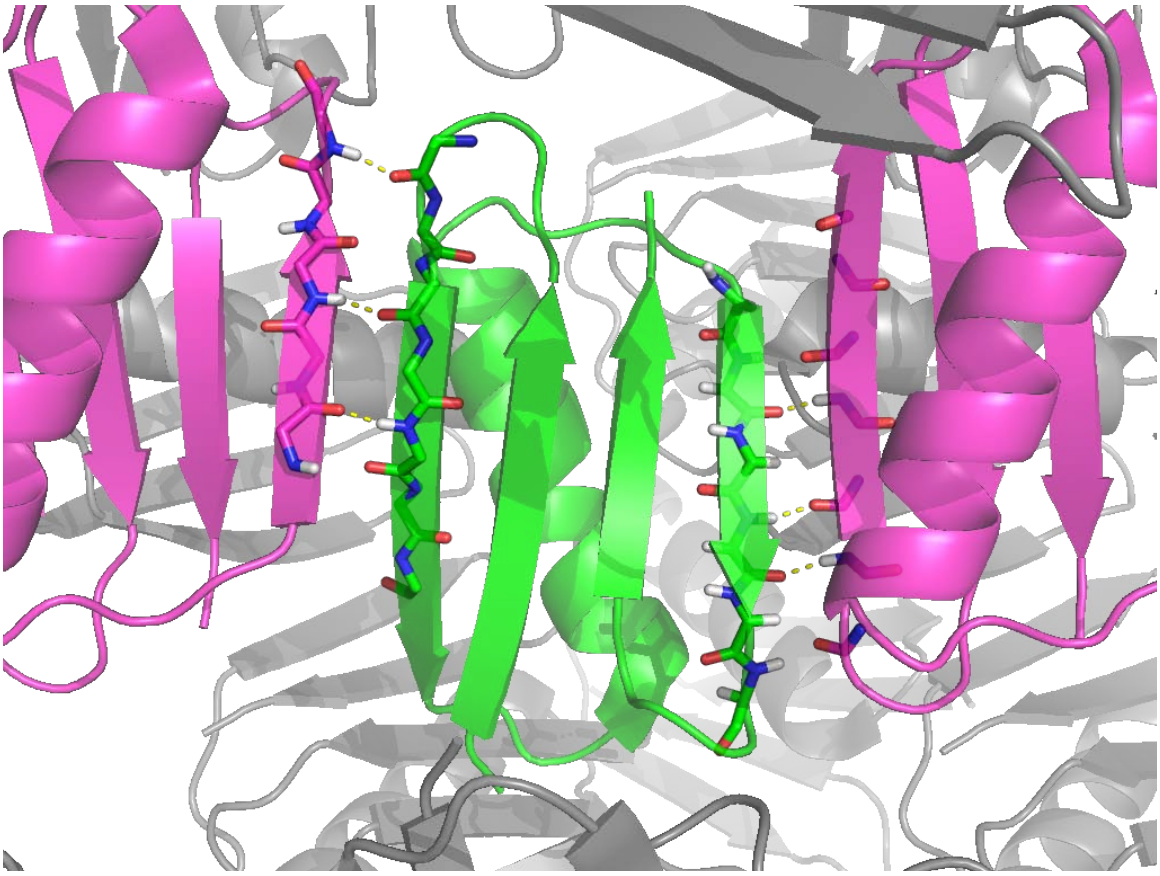

Figure S5. Intermolecular H-bonds in the GB1 crystal. The picture was generated using the Protein Data Bank file 2QMT.

References

Krushelnitsky A, Zinkevich T, Reif B, Saalwächter K (2014) Slow motions in microcrystalline proteins as observed by MAS-dependent ^{15}N rotating-frame NMR relaxation. J Magn Reson 248:8-12.

Kurbanov R, Zinkevich T, Krushelnitsky A (2011) The nuclear magnetic resonance relaxation data analysis in solids: General $R_1/R_{1\rho}$ equations and the model-free approach. J Chem Phys 135:184104.

Lamley JM, Oster C, Stevens RA, Lewandowski JR (2015) Intermolecular interactions and protein dynamics by solid-state NMR spectroscopy. Angew Chem Int Ed. 51:15374-15378.

Rovo P, Linser R (2017) Microsecond time scale proton rotating-frame relaxation under magic angle spinning. J Phys Chem B 121:6117-6130.

Schmidt HLF, Sperling LJ, Gao YG, Wylie BJ, Boettcher JM, Wilson SR, Rienstra CM (2007) Crystal polymorphism of protein GB1 examined by solid-state NMR spectroscopy and X-ray diffraction. J Phys Chem B 111:14362-14369.

Schneider H (1991) Modified Fuoss-Kirkwood distribution function for interpretation of asymmetric NMR relaxation curves. J Polym Sci B 29:1171-1177.

Zhou DH, Shea JJ, Nieuwkoop AJ, Franks WT, Wylie BJ, Mullen C, Sandoz D, Rienstra CM (2007) Solid-state protein-structure determination with proton-detected triple-resonance 3D magic-angle-spinning NMR spectroscopy. Angew Chem Int Ed 46:8380-8383.
